# Digital Intervention for Psychedelic Preparation (DIPP): protocol for a randomised controlled feasibility trial comparing meditation- and music-based programmes in healthy volunteers

**DOI:** 10.1136/bmjopen-2025-107512

**Published:** 2026-03-12

**Authors:** Rosalind McAlpine, Magdalena Jaglinska, Krisztina Jedlovszky, Joanna Kuc, Ariel Castro, Alexandre Piot, Christopher Timmermann, Jeremy I Skipper, Matthew D Sacchet, Sunjeev K Kamboj

**Affiliations:** 1 Clinical, Educational and Health Psychology, University College London, London, UK; 2 Experimental Psychology, University College London, London, UK; 3 Psychological Sciences, Birkbeck University of London, London, UK; 4 Centre for Psychedelic Research, Imperial College London, London, UK; 5 Harvard Meditation Research Program, Massachusetts General Hospital, Boston, Massachusetts, USA

**Keywords:** Telemedicine, Clinical Protocols, PSYCHIATRY, Randomized Controlled Trial

## Abstract

**Introduction:**

Psychedelic-assisted therapy shows promise for treating various mental health conditions; however, its reliance on intensive psychological preparation limits its broader application. Digital health interventions have the potential to address this limitation by providing structured, accessible and scalable preparation solutions. This randomised controlled feasibility trial aims to evaluate the feasibility and preliminary efficacy of the Digital Intervention for Psychedelic Preparation (DIPP), a 21-day mobile-accessible programme designed to prepare individuals for psychedelic experiences.

**Methods and analysis:**

The study will recruit 40 non-treatment-seeking adults without a clinical diagnosis, randomly assigning them to one of two conditions: (1) DIPP-MEDITATE, which combines daily guided meditation with background music or (2) DIPP-MUSIC, which provides the same background music without guided meditation. Both groups will complete the 21-day digital intervention remotely. Following the intervention, participants will attend an in-person supervised psilocybin session, receiving a standardised 25 mg dose. Primary outcomes focus on feasibility metrics including recruitment efficiency, participant retention and adherence to the intervention protocol. Secondary outcomes assess subjective feasibility, acceptability and preliminary efficacy, specifically evaluating psychedelic preparedness, the quality of the psychedelic experience and changes in wellbeing, with follow-up assessments at 2 weeks, and at 3, 6 and 9 months post-session. Exploratory measures include neuroimaging, physiological, cognitive and psychological assessments, as well as voice note experience sampling through a chatbot (referred to as ‘DIPP-bot’) to monitor inner speech, thought and emotional states during the intervention and follow-up periods.

**Ethics and dissemination:**

Approved by UCL Research Ethics Committee (ID: 19113/003), this study follows the Declaration of Helsinki. Results will be published in peer-reviewed journals and presented at conferences. Confidentiality will be maintained throughout.

**Trial registration number:**

NCT06815653.

**Strengths and limitations of this study**

- Participants remain blind to condition, study design (ie, randomisation to DIPP-MEDITATE/MUSIC conditions) and study hypotheses.
- The 21-day mobile-accessible programme is scalable, beginner-friendly and requires no prior experience, supporting broad accessibility.
- Comprehensive data collection, including self-report, neurophysiological and behavioural measures, allows for a multidimensional evaluation of intervention effects.
- Application to specific diagnoses remains uncertain.
- The study lacks a non-digital control group, preventing direct comparisons between digital and traditional psychedelic preparation methods.

## Introduction

 Classic serotonergic psychedelics such as psilocybin, lysergic acid diethylamide (LSD) and N,N-dimethyltryptamine (DMT) are gaining significant attention in mental health research for their potential to treat conditions such as depression, anxiety and substance use disorders.^1^,^5^ These compounds induce profound shifts in consciousness^6 7^ and can lead to lasting changes in beliefs, attitudes and behaviours.^8^,^12^ However, the same intensity that underpins their therapeutic potential may present substantial psychological risks, including acute anxiety, psychological distress and, in rare cases, prolonged psychotic reactions.^13^,^15^ To address these challenges, protocols for clinical trials of psychedelic substances emphasise comprehensive psychological preparation as a cornerstone component of psychedelic interventions,^16^ incorporating psychoeducation, intention-setting practices and emotional regulation training.^17 18^ While these preparation frameworks effectively establish safety parameters and create a therapeutic container for processing challenging experiences, they typically require intensive therapist engagement. This represents a significant barrier to scalability as psychedelic interventions move towards larger clinical trials and, potentially, integration into healthcare systems. A key challenge moving forward is determining which preparation components can be adapted for scalable delivery without compromising their psychological depth, experiential impact or efficacy (the capacity to mitigate risks and/or enhance therapeutic impact). Importantly, increasing the role of self-guided approaches may not only support broader access but also reduce the risk of boundary violations associated with therapist-led preparation,^19 20^ further enhancing safety alongside scalability. However, it remains critical that core elements—such as psychoeducation, intention-setting and emotional regulation—retain their full integrity, even when delivered with reduced resources.

Digital health interventions (DHIs) offer a scalable solution to addressing these implementation challenges. Several systematic reviews and meta-analyses have demonstrated that DHIs can achieve comparable outcomes to traditional face-to-face therapy across multiple mental health conditions, while substantially reducing resource requirements and expanding access to care.^21^,^23^ In the context of psychedelic-assisted therapy (PAT), digital tools are increasingly being discussed as potential ways to enhance treatment. These include virtual reality for immersive therapeutic environments, biofeedback and wearable devices for real-time physiological monitoring, Artifical Intelligence (AI)-driven platforms for personalised treatment delivery and optimisation, and smartphone applications for outcome tracking and integration support.^24^,^26^ However, their application to psychedelic preparation remains largely unexplored, representing a crucial gap in the literature that warrants systematic investigation.

**Figure 1:**
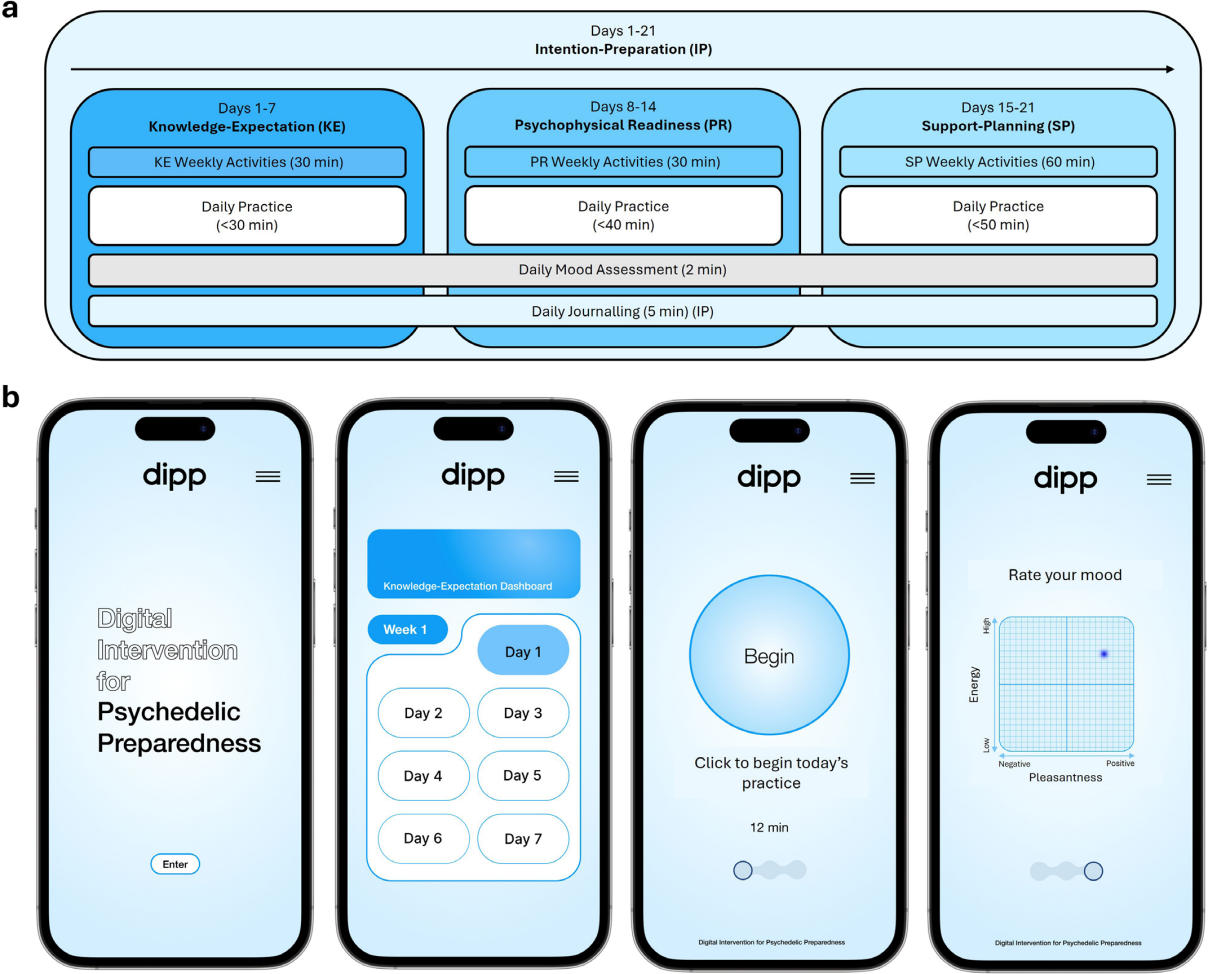
Structure and interface of the Digital Intervention for Psychedelic Preparation (DIPP). (**a**) Overview of the 21-day programme structure, organised around the four-factor model of psychedelic preparedness: Knowledge-Expectation (KE), Psychophysical Readiness (PR), Intention-Preparation (IP) and Support-Planning (SP). Daily activities comprise a practice session (ambient music in DIPP-MUSIC; ambient music plus guided meditation in DIPP-MEDITATE), mood tracking, and journalling. Weekly activities progressively introduce content relevant to each preparation factor. (**b**) Screen images from the DIPP mobile application, showing from left to right the home screen, KE dashboard, daily practice interface, and mood tracking tool. (Figure adapted from McAlpine *et al*^17^).

To address this gap, we developed the Digital Intervention for Psychedelic Preparation (DIPP),^27^ a 21-day self-guided programme structured around a four-factor model of psychedelic preparedness^17^ (figure 1a). The model includes: Knowledge-Expectation (KE), which refers to understanding the nature of psychedelic experiences and the possibility of intense or unpredictable effects; Psychophysical Readiness (PR), which encompasses psychological and bodily readiness to face challenging emotions and sensations; Intention-Preparation (IP), which involves reflecting on personal motivations and clarifying goals for the experience and Support-Planning (SP), which focuses on establishing a safe interpersonal and physical environment before, during and after the experience, including coping strategies and integration planning. Each day, participants engage in a daily practice, mood tracking and journalling, with journalling specifically supporting IP by assisting participants in clarifying their motivations and setting intentions. In addition to these daily components, participants complete self-paced weekly activities tailored to each preparation factor: KE (educational content, week 1), PR (grounding and embodiment exercises, week 2) and SP (support system development, coping strategies and integration planning, week 3). The intervention is delivered via a dedicated web application (figure 1b), ensuring accessibility and flexibility. While initially designed for psilocybin, DIPP’s adaptable structure allows modification for use with other psychedelics.

A core component of DIPP is a daily practice lasting 20–50 minutes, with duration progressively increasing over the 21-day programme. In the **DIPP-MEDITATE** programme, this consists of guided Loving-Kindness Meditation (LKM) delivered over ambient music; in the **DIPP-MUSIC** programme, participants receive the same ambient music without guided meditation instruction (see *Trial Objectives* below). Our previous study, which surveyed meditation practitioners with experience of psychedelic drug use, identified Loving Kindness Meditation (LKM) (inspired by the Theravada Buddhist practice of *mettā bhavana* (in Pāli, the liturgical language))^28 29^ as the most effective practice for psychedelic preparation, significantly outranking meditation approaches based on Focused Attention and Open Monitoring.^30^ Accordingly, LKM serves as the primary meditation technique in DIPP-MEDITATE. The programme systematically cultivates loving-kindness (*mettā*) in three stages: first toward oneself (days 1–7), then extending to another person (days 8–14), and finally expanding outward to encompass all beings (days 15–21).^31^,^33^ Alongside this, participants are introduced to structured methods for working with distractions and refining their meditation practice,^32 33^ including the ‘6Rs’ technique (Recognise, Release, Relax, Re-smile, Return, Repeat)—a cyclical process designed to disengage from distractions in a gentle, non-forceful manner^34 35^—and a framework for recognising different levels of awareness^36 37^ (see online supplemental S1 and S2 for details). These elements support participants in cultivating attentional stability, increasing clarity of awareness and fostering insight into their mental processes,^38^,^40^ thus equipping them with useful skills for navigating shifts in consciousness during psychedelic experiences. A separate publication will provide a detailed account of the meditation framework supporting the DIPP-MEDITATE programme.

In addition to the primary feasibility outcomes and self-report measures, the trial includes several exploratory components designed to capture dynamic psychological and physiological changes that traditional questionnaires may overlook. First, a Telegram-based chatbot (DIPP-bot) facilitates thought sampling, prompting participants to submit brief (~1 min) voice notes and structured follow-up questions at semi-random intervals during the 10 days before and after dosing, and at 3, 6 and 9 months post-dosing. Voice notes capture both semantic content and paralinguistic features (eg, speech rate, pitch variability) that may index emotional and cognitive states, allowing for examination of how inner experience and mental content shift across the psilocybin experience^41^,^43^ (see online supplemental S7 for DIPP-bot architecture and prompts). Second, naturalistic functional MRI (fMRI; hereafter movie-fMRI) is conducted one day before dosing and 2 weeks after, with participants viewing a feature film to assess whether the intervention and psilocybin session are associated with changes in whole-brain functional network organisation^44^,^47^; standard MRI eligibility criteria are therefore included in this protocol. Third, continuous physiological monitoring using a WHOOP wearable device and salivary cortisol sampling enables examination of whether meditation-based preparation influences stress-related markers (Heart Rate Variability (HRV), sleep, cortisol) and allows control for potential confounders. Fourth, a battery of cognitive tasks is administered at key timepoints to explore potential changes in cognitive function. As these components are exploratory, detailed methods and analyses will be presented in separate publications. A complete overview of all outcome measures, including additional self-report measures, is provided in online supplemental S4 and S5.

### Trial objectives

**Figure 2:**
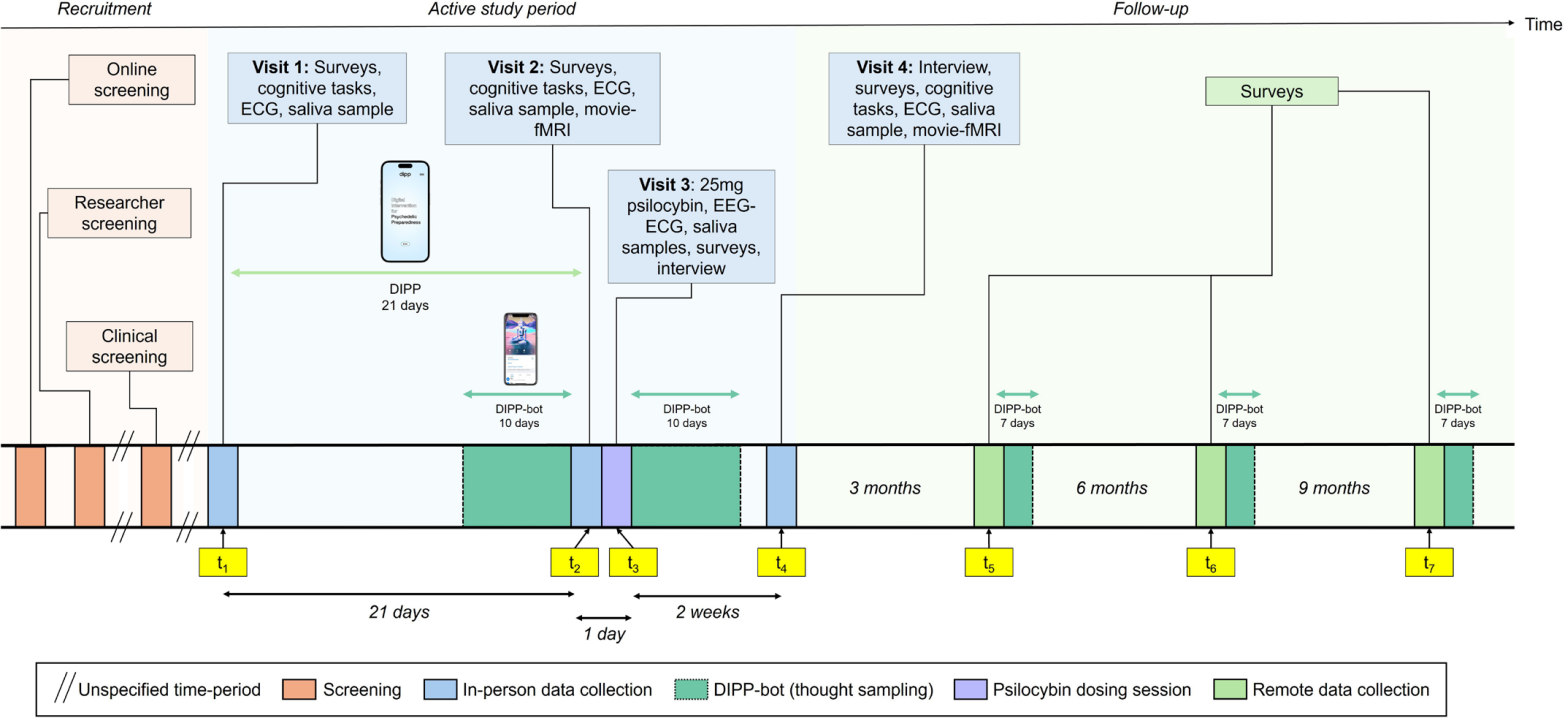
Study timeline. Screening activities are shown in orange, in-person data collection (including key study visits) in blue, and remote data collection in green. The purple rectangle represents the psilocybin dosing session. DIPP-bot thought sampling periods are indicated in dark green with dashed outline. Participants are randomised to either DIPP-MEDITATE or DIPP-MUSIC for the 21-day pre-dosing intervention period. Key assessment timepoints are labelled t₁–t₇. ECG, electrocardiogram; EEG, electroencephalogram; movie-fMRI, movie-viewing functional magnetic resonance imaging; DIPP-MEDITATE, Digital Intervention for Psychedelic Preparedness – Meditate; DIPP-MUSIC, Digital Intervention for Psychedelic Preparedness – Music.

To evaluate whether guided meditation substantially contributes to the acceptability, feasibility, and preliminary efficacy of DIPP, we designed a randomised controlled feasibility trial following Standard Protocol Items: Recommendations for Interventional Trials (SPIRIT) guidelines^48^ (SPIRIT table and checklist can be found in online supplemental S4). Forty healthy volunteers will be randomised to DIPP-MEDITATE or DIPP-MUSIC. The trial timeline comprises: baseline (t_1_), pre-dosing (t_2_, day 21), dosing (t_3_, day 22), two-week follow-up (t_4_), and remote follow-ups at 3, 6, and 9 months post-dosing (t_5_–t_7_) (see figure 2).

The DIPP-MUSIC control was designed to control for attentional aspects, intervention duration, digital engagement/sensory input, and to isolate the effects of LKM. As such, participants in both groups will engage with the same digital platform, follow the same schedule and receive the same preparatory educational materials, ensuring that any observed differences are specifically attributable to the guided meditation component rather than general exposure to a structured preparation programme. Importantly, the two conditions are fully matched on exposure time: the DIPP-MUSIC audio presents the same ambient music for the full session duration, with only the guided meditation instructions removed. By using the same ambient audio in both conditions, we control for potential effects of music while removing explicit meditation guidance. Crucially, study information simply refers to ‘preparation activities’ without specifying these in detail or mentioning the presence of multiple groups. This was done to ensure expectancy effects could not differ prior to starting the DIPP (± guided meditation) interventions.

Following the 21-day preparation period, participants will undergo a supervised 25 mg psilocybin session at University College London (UCL) (t_3_). Follow-up assessments will be conducted in person at 2 weeks (t_4_) and online at 3, 6 and 9 months postintervention (t_5-7_). The trial will primarily assess operational feasibility and intervention adherence, while secondary outcomes will evaluate implementation metrics and preliminary efficacy measures.

## Methods and analysis

### Recruitment

Forty non-treatment-seeking healthy adults residing in the UK will be recruited through institutional mailing lists, social media platforms and word-of-mouth referrals. Written informed consent will be obtained from all participants prior to enrolment (online supplemental S3). Participant recruitment will take place between June 2025 and June 2026. Data collection, including the final 9-month follow-up assessments, is expected to continue through to September 2027.

### Sample size

Because this is a feasibility trial, no formal power calculation was conducted. A sample of 40 participants (20 per group) was chosen based on published guidance for early-phase trials, ensuring sufficient data to assess recruitment, retention and adherence.^49^,^51^

### Patient and public involvement

Patient and public involvement (PPI) was central to the intervention’s development, following co-creation principles that prioritise working with, rather than for, individuals with lived experience.^52^,^54^ This systematic approach, aligned with best practices in digital mental health^21 55 56^ and psychedelic research,^57^,^59^ ensured PPI at multiple stages. Guided by Medical Research Council recommendations,^60^ the process was dynamic and iterative, integrating PPI to shape both core components and delivery methods.

The PPI strategy consisted of two complementary components. First, we conducted in-depth qualitative interviews with 19 participants who had previously received high-dose psilocybin in retreat settings.^27^ These semi-structured interviews systematically explored preparation behaviours, intervention preferences and perspectives on digital delivery methods. Insights from these interviews directly informed the core intervention components and structure. Building on these initial findings, we then conducted three iterative co-design workshops with a different set of 28 psilocybin retreat participants.^27^ These workshops facilitated collaborative refinement of the intervention through structured activities, feasibility assessments and collective problem-solving sessions. Participants evaluated potential benefits, risks and ideal outcomes of key intervention features, generating practical solutions grounded in their lived experience. This iterative process allowed us to identify and address potential implementation barriers early in the development phase.

Participant recommendations across both phases informed multiple aspects of the intervention design. The selection and integration of core components (eg, meditation practices, educational resources, grounding techniques) were driven by expressed user needs and preferences. Programme structure was similarly shaped by participant input, determining the duration (21 days), daily time commitment (mean recommendation of 36 min) and modular organisation. Participant preference (63% favouring online delivery) supported the digital format implementation, informing the integration of interactive elements and flexible engagement options.

A PPI advisory group comprising four individuals who previously participated in a PAT trial has been established to guide trial implementation. This group has reviewed recruitment materials and strategies to ensure they resonate with potential participants. They have also assessed the burden of participation, including the time commitments for both the digital intervention and follow-up assessments. We will continue PPI throughout this feasibility trial. For results dissemination, the group will help develop accessible summaries of the findings for participants and the wider psychedelic research community, determining the most effective formats for communicating various outcomes.

### Eligibility criteria

Participants must be aged between 21 and 65 years and have limited prior psychedelic use, defined as five or fewer ‘full-dose’ experiences (eg, ≥2g of dried psilocybin mushrooms or equivalent) and none within the past 6 months. Microdosing, defined as the repeated use of sub-perceptual doses that do not produce noticeable alterations in consciousness, is not counted as a full-dose experience.^61^ Participants must also have minimal meditation experience, defined as no more than ten sessions exceeding 30 min, and no prior retreats or regular ongoing practice. This criterion is intended to reduce variability in baseline meditation ability and ensure that any observed effects on psychedelic preparedness can be more confidently attributed to the intervention itself. Additional eligibility requirements include being a native English speaker, having normal or corrected-to-normal colour vision, and being able and willing to provide informed consent. Participants must be UK residents registered with a primary care provider, have access to a smartphone and be able to comply with all study requirements, including both in-person and remote sessions. They must also agree to allow the research team to contact their primary or secondary care providers, if necessary, identify a support person for post-session accompaniment, provide emergency contact details and maintain access to an electronic device for data entry.

Individuals will be excluded if they have any current or past diagnosis of a psychiatric disorder. An exception may be made for individuals with a history of a mood disorder (eg, a past depressive episode) provided that the episode has been in stable remission for at least 5 years and the clinical team determines that participation poses minimal risk. Those with a current or past diagnosis of a psychotic or bipolar disorder, or an immediate family history of these conditions, are not eligible under any circumstances. Additional exclusion criteria include a history of serious suicidal ideation (eg, intent or planning) or any past suicide attempts. Participants must not have medically significant physical health conditions that could pose a risk with psilocybin or MRI, such as cardiovascular disease, uncontrolled hypertension, epilepsy, migraines or focal scalp sensitivity. Use of medications that interact with psilocybin, including antipsychotics, selective serotonin reuptake inhibitors, serotonin-norepinephrine reuptake inhibitors, tricyclic antidepressants and mood stabilisers, is also a disqualifier. Participants must not have used psychoactive drugs within 30 days of enrolment, excluding alcohol, nicotine and caffeine. Furthermore, individuals who are pregnant, planning a pregnancy or breastfeeding will not be eligible. Those who have participated in a drug trial within the past 6 months or have MRI contraindications (eg, metal implants, pacemakers or severe claustrophobia) are also excluded from the study.

### Screening

All prospective participants will be directed to complete an online pre-screening questionnaire which contains a list of questions on demographics, physical and mental health, substance use and other factors essential for assessing initial eligibility. Those who meet the initial criteria will be invited to a remote screening call with a member of the research team, where they will receive detailed information about the study and can ask questions before electronically providing informed consent. The remote screening call will also include a psychiatric evaluation using the Mini-International Neuropsychiatric Interview,^62^ the Standardised Assessment of Personality^63^ to screen for personality disorders, and the Stressful Life Events Screening Questionnaire^64^ to evaluate exposure to traumatic events. Participants who pass this stage will be invited to a more comprehensive online clinical interview with a licensed clinical psychologist to confirm their eligibility and provide further clarification about study procedures. Resting blood pressure will be measured in person during the baseline visit (t_1_) to assess cardiovascular suitability for psilocybin administration. Participants with abnormally elevated blood pressure (above 130/80 mmHg) may be excluded from the study at the discretion of the clinical team.

### Allocation methods and blinding

Participants will be randomly assigned in a 1:1 ratio to one of two study arms (DIPP-MEDITATE or DIPP-MUSIC) using a random number generator prepared prior to trial commencement. The allocation list will be stored in a secure, password-protected file inaccessible to researchers involved in data collection. A researcher with no involvement in these processes will inform the lead researcher of each participant’s allocation on arrival to enable setup of the appropriate version of the programme.

To minimise expectancy effects, participants are not informed that the study involves randomisation or that there are different versions of DIPP. During the consent process, participants are told they will complete a 21-day digital preparation programme involving daily audio-based activities; no reference is made to different conditions or to the presence of a control group. This incomplete disclosure is necessary to ensure that any observed differences between groups can be attributed to the intervention itself rather than participant expectations. At study completion (t_7_), participants are fully debriefed and informed of the study design and their allocated condition. This procedure was reviewed and approved by the UCL Research Ethics Committee.

Both interventions are matched in structure, interface and schedule. The only difference is the presence or absence of guided meditation within the audio content. All participants will receive standardised instructions (see online supplemental S6) and complete the Credibility and Expectancy Questionnaire^65^ at baseline (t_1_). At the final study visit (t_4_), participants will be asked whether they believe they received the ‘complete DIPP programme’, to assess perceived intervention exposure. As both interventions are fully automated and data will be self-reported, there is no opportunity for researcher influence on outcomes. Any procedural errors leading to inadvertent disclosure of group assignment will be recorded and reported.

### Design

Participants will complete a structured 5-week protocol involving four in-person study visits: baseline (t_1_), predosing (t_2_), dosing (t_3_) and postdosing follow-up (t_4_) (see figure 2). At t_1_, they will complete a battery of self-report questionnaires and cognitive tasks assessing psychological wellbeing and inner experience, alongside a 5-minute resting electrocardiogram (ECG) and a saliva sample for cortisol analysis. Participants will be issued a WHOOP wearable device to continuously track biometric data through to t_4_. Between t_1_ and t_2_, participants will engage in the 21-day DIPP. During the 10 days before and after dosing, they will also provide daily voice note samples via the DIPP-bot mobile app for linguistic and acoustic analysis. Further details on the prompt structure and DIPP-bot architecture are provided in online supplemental S7. At t_2_, participants will repeat the battery of questionnaires and cognitive tasks, ECG and saliva sampling from t_1_, and complete a movie-fMRI scan. One day later at t_3_, they will receive a 25 mg dose of psilocybin, with electroencephalogram (EEG) and ECG recorded at baseline (~15 minutes before drug administration), 90 min and 150 min postadministration. Saliva samples will be collected at the same time points for cortisol analysis. At the end of the dosing session, participants will also complete a semi-structured qualitative interview and a battery of validated questionnaires assessing subjective drug effects and phenomenological aspects of the experience**.** At t_4_, participants will complete a semi-structured interview, followed by the battery of questionnaires and cognitive tasks, ECG and saliva sample, and undergo a second movie-fMRI scan. They will also return the WHOOP device, concluding continuous biometric tracking. Remote follow-ups will continue for 9 months, with online assessments and renewed thought sampling via DIPP-bot at 3 (t_5_), 6 (t_6_) and 9 (t_7_) months postdosing.

### Outcome measures

The study employs a comprehensive, multimodal data collection approach, incorporating self-report measures, neurophysiological assessments, behavioural indices and qualitative data. The primary outcomes focus on key indicators of feasibility and adherence, including recruitment efficiency, study retention and engagement (intervention adherence) with DIPP. Recruitment efficiency is measured as the weekly rate of participant enrolment, calculated as the total number of participants successfully completing online screening, researcher screening and clinical screening, divided by the total number of recruitment weeks. Acceptable recruitment efficacy is defined as maintaining an average recruitment rate of at least one participant per week until the target sample of 40 participants is reached. This measure is tracked from study initiation to the completion of recruitment. Study retention is evaluated as the percentage of participants who complete the 2-week post-dose follow-up assessment (t_4_). Acceptable study retention is defined as at least 70% of participants completing the (t_4_) assessment. Intervention adherence will be assessed based on completion of three daily tasks (practice (± guided meditation), mood rating and journal entry) and 2-weekly module-specific tasks, totalling 69 tasks over the 21-day programme (63 daily+6 weekly). Individual adherence is defined as completion of ≥48 tasks (≥70%). Intervention adherence success is defined as ≥70% of participants meeting this individual adherence threshold.^66^ A complete overview of all outcome measures is presented in a SPIRIT table in online supplemental S4, with primary, secondary and other prespecified outcomes detailed in online supplemental S5.

### Analysis strategy and missing data

This section outlines the analysis plan for primary and secondary outcomes. Exploratory outcomes, including behavioural and neurophysiological measures, will be detailed in separate publications.

Primary feasibility outcomes (recruitment efficiency, study retention and intervention adherence) will be summarised as frequencies and proportions.

Secondary outcomes include a custom Subjective Feasibility of Intervention Scale (SFIS), the Theoretical Framework of Acceptability (TFA),^67^ the System Usability Scale (SUS),^68^ the Mobile Application Rating Scale (MARS),^69^ the Psychedelic Preparedness Scale (PPS),^17^ the Altered States of Consciousness Rating Scale (5D-ASC),^70^ the Challenging Experience Questionnaire (CEQ-7)^71^ and the Short Warwick-Edinburgh Mental Wellbeing Scale (SWEMWBS).^72^ As this feasibility study is not powered to detect between-group differences, analyses are descriptive and exploratory rather than confirmatory. Subjective feasibility and acceptability measures (SFIS, TFA, SUS, MARS) will be summarised descriptively by condition. Linear mixed models will be used to examine change over time for repeated measures (PPS, SWEMWBS), and independent t-tests to compare groups on acute measures (5D-ASC, CEQ-7). Effect sizes and 95% CIs will be reported; p-values may be presented descriptively but will not be used for inference.

For primary outcomes, all available data will be included with no imputation. Linear mixed models accommodate missing data under missing-at-random assumptions. Patterns of missingness will be assessed and reported.

### Investigational medicinal product management

The study uses Good Manufacturing Practice (GMP)-grade psilocybin manufactured and encapsulated by Filament Health (PEX0101, Psilo Scientific). All handling and storage procedures comply with Schedule 1 controlled substance regulations under a UK Home Office licence. GMP standards are maintained throughout the manufacturing and storage process. The investigational medicinal product (IMP) is stored in a secure controlled-drug facility at the Clinical Psychopharmacology Unit, UCL, with strict access controls and documentation procedures in place.

### Data monitoring

An independent Data Monitoring Committee has not been convened for this study. This decision is based on several factors: the small sample size (n=40), single-dose administration of the IMP, recruitment of healthy volunteers rather than a clinical population, and the absence of planned interim efficacy analyses. Psilocybin has an established safety profile at the administered dose (25 mg) in supervised research settings, and all dosing sessions are conducted under medical oversight with standardised safety protocols. This approach is consistent with proportionate risk-based monitoring guidance for early-phase experimental medicine studies.

Data monitoring will be conducted internally by the principal investigator (PI) and designated team members who are independent from day-to-day delivery of the intervention. Their role is to oversee adherence to the approved protocol, ensure data integrity and protect participant safety. No interim analyses are planned, and the study is not designed with formal stopping rules for efficacy. Monitoring of trial conduct will be conducted via scheduled internal audits, including monthly checks of consent documentation, data completeness and adverse event (AE) logs. Any potential conflicts of interest are managed in accordance with the host institution’s ethics policies, and any issues that arise will be reviewed promptly by study leadership. These procedures are proportionate to study risk and ensure ongoing compliance with relevant ethical and regulatory standards.

### Safety monitoring

AEs will be monitored and documented throughout the study. The clinical team will categorise AEs based on severity (mild: no impact on daily activities; moderate: some interference with daily activities; severe: prevents daily activities), whether they are anticipated or unanticipated, and their causal relationship to the IMP (classified as related, possibly related or unrelated as determined by a supervising medical doctor). AE summary tables will present the total number of participants reporting an AE, the percentage of participants affected, and the total number of events recorded. Serious AEs (SAEs) and Serious Adverse Reactions (SARs) will be defined as any significant medical occurrence that (1) leads to death, (2) poses a life-threatening risk, (3) necessitates hospitalisation or (4) results in long-term disability or impairment. All SAEs and SARs will be promptly reported to the PI immediately on the study team becoming aware of the incident. The research ethics committee will also be alerted as soon as any such reaction is detected.

### Psychological support

#### Preparation

This study employs researchers with prior psychedelic trial experience who serve as ‘guides’ during dosing sessions (t_3_). All guides receive an induction led by the study’s primary clinical psychologist, covering key elements of psychedelic support, safety protocols and participant care, with regular supervision throughout the study. Two guides are assigned to each participant and accompany them throughout the trial, ensuring physical and psychological safety while remaining neutral to the content of the participant’s experience. This approach aligns with standardised practices in psychedelic research^73^ while distinguishing the support model from therapeutic intervention.

Direct contact with guides is intentionally limited, as the majority of preparation is delivered remotely via the 21-day DIPP platform (t_1–2_).^27^ In-person guide contact comprises three touchpoints: At baseline (t_1_), guides meet briefly with participants (~30 min) to cover psychoeducation, build rapport and discuss intention setting. The day before dosing (t_2_), participants return for a final preparation session (~30–45 min) to discuss dosing-day preferences (eg, handholding, room set-up) and review individual coping strategies developed through DIPP. On the dosing day (t_3_), guides remain present throughout to ensure safety and provide support as needed.

#### Dosing session

Following established research protocols,^74^ the guides maintain a non-directive, person-centred approach that allows participants to process their experiences autonomously. Participants will spend approximately 8 hours at the research facility on their dosing day (t_3_). The acute drug effects typically last 4–6 hours. Sessions will be conducted in a comfortable room with adjustable, low lighting. Participants will be provided with an eye mask and headphones, which they can use according to their comfort level, and will be encouraged to maintain a semi-reclined position when possible. The session will include a carefully curated music playlist, developed in collaboration with music therapists. A qualified medical professional will be present on-site throughout the session to monitor participants’ safety and wellbeing. Before discharge, the research team will conduct a sobriety assessment (including the Drug Effects Questionnaire^75^ and the simplified six-Item clinician administered dissociative symptom scale)^76^ to ensure participants are fit to leave. All participants must be accompanied home by a trusted companion after the session.

#### Integration

During the SP module of DIPP, completed in the week prior to dosing, participants create a personalised ‘integration plan’ to support processing of their upcoming psychedelic experience. They are guided through a structured framework to reflect on potential insights, identify areas for personal growth and select strategies for integration, including emotional processing, reflection and behavioural change. The instructions and format are implemented consistently across both conditions; however, the content of each plan is participant-generated, introducing some variability in postdosing behaviours that may influence longer-term outcomes. Nonetheless, integration planning is considered an essential part of the intervention, included for ethical and safety reasons.

The day after dosing (t_3+1_), participants receive an email check-in from their guides to address any immediate reflections or concerns. A follow-up call (~1 hour) is available on request. Two weeks postdose (t_4_), participants attend an in-person integration session with their guides, lasting 30–60 min, where they reflect on their experience, discuss emerging insights and explore how to apply them in daily life. This session also includes standardised self-report assessments as part of study data collection.

## Ethics and dissemination

### Dissemination

Study findings will be disseminated through multiple channels to maximise scientific impact and public benefit. Primary outcomes will be published in peer-reviewed academic journals and presented at relevant scientific conferences. To enhance public understanding and engagement with the research, findings will also be communicated through appropriate public forums and media channels. All dissemination activities will maintain strict participant confidentiality and anonymity. The PI will oversee all publications and presentations. Any parallel studies utilising study data will follow international authorship guidelines (eg, International Committee of Medical Journal Editors (ICMJE) criteria) while acknowledging the Study Coordination Team’s contribution to the primary research programme.

### Data management

Data management procedures follow UCL’s Standard Operating Procedures and a study-specific data management plan. All voice note samples, follow-up responses and engagement logs collected via the DIPP-bot are securely stored on Amazon Web Services servers, managed through OneReach.AI. All data collection and storage systems comply with General Data Protection Regulation (GDPR) requirements and have received approval from UCL’s Information Security team.

### Ethical approval

This study has received full approval from the UCL Research Ethics Committee (UCL REC; ID: 19113/003) and will be conducted in accordance with the Declaration of Helsinki and relevant UK research governance frameworks. Any important protocol modifications (eg, changes to eligibility criteria, outcomes or procedures) will be submitted to the UCL REC for approval before implementation and communicated to relevant parties, including the sponsor, collaborators and trial registries. Participants will be reconsented if modifications affect their participation or the use of their data. Written informed consent will be obtained remotely by trained research staff prior to any study procedures, with opportunities for participants to ask questions and receive verbal checks of understanding. There are no ancillary studies or plans for biological specimen collection at this time; should such components be introduced, separate ethical approval and consent will be obtained. Confidentiality will be strictly maintained, with personal information stored separately from deidentified data on secure, access-controlled servers in compliance with GDPR and UCL policies. Identifiable data will be deleted after study completion, and anonymised data will be retained in accordance with the study’s Data Management Plan. As this is a non-clinical experimental medicine study involving healthy volunteers with comprehensive safety protocols in place, no ancillary or post-trial care beyond the structured integration support described above is planned; however, participants will have continued access to the research team. In the unlikely event of study-related harm, participants will be covered by UCL’s institutional insurance policy, and AEs will be managed in line with sponsor and ethics procedures. Participants may request to discontinue or withdraw from the intervention at any time without providing a reason. The allocated intervention may also be modified or discontinued at the discretion of the clinical team if AEs, emerging medical concerns or participant distress indicate that continuation would no longer be in their best interest.

## Supplementary material

10.1136/bmjopen-2025-107512 online supplemental file 1
