# Supplementary material for: Digital Intervention for Psychedelic Preparation (DIPP): protocol for a randomised controlled feasibility trial comparing meditation- and music-based programmes in healthy volunteers
Source: BMJ Open. 2026 Mar 12;16(3):e107512. doi: 10.1136/bmjopen-2025-107512 (PMC12983862; doi:10.1136/bmjopen-2025-107512)
Supplement: online supplemental file 1 [file bmjopen-16-3-s001.docx]

Supplementary Material: Digital Intervention for Psychedelic Preparation (DIPP): Protocol for a randomised controlled feasibility trial comparing meditation and non-meditation programs in healthy volunteers

**S1 The 6Rs method**

*Overview*

The 6Rs is a structured method designed to help meditators recognise and skillfully work with distractions. This approach is central to the Digital Intervention for Psychedelic Preparation (DIPP), as it provides a systematic way to disengage from distractions while cultivating a relaxed and open awareness. Originally developed as part of the Tranquil Wisdom Insight Meditation (TWIM) system, the 6Rs method is rooted in early Buddhist teachings and was adapted at the Dhamma Sukha Meditation Center as a contemporary guidance system for mindfulness training.

The 6Rs cycle consists of six sequential steps that allow practitioners to navigate their meditation experience effectively without force or suppression. This process is particularly relevant for psychedelic preparation, where developing non-reactivity, equanimity, and attentional flexibility is essential for navigating altered states of consciousness.

*Step-by-step breakdown of the 6Rs*

1. Recognise (Pali: *Janati* - जानाति): The first step is to recognise that the mind has wandered. This requires mindfulness (*sati)* - the ability to observe how attention moves from moment to moment. Distractions may arise in the form of thoughts, emotions, physical sensations, or external stimuli. Recognising them as distractions is the first step in working with them skillfully.
   1. Relevance to Psychedelic Preparation: Recognising shifts in attention is crucial for navigating the unpredictable nature of psychedelic experiences. Training in this skill helps participants notice and engage with arising phenomena without becoming overwhelmed or reactive.
2. Release (Pali: *Vineyya* - विनेय्य): After recognising a distraction, the next step is to release it - allowing it to be without engaging further. This is done gently, without force, by letting go of any attachment to the distraction. The content of the thought, emotion, or sensation is not important; what matters is the ability to recognise its impermanent nature and avoid grasping onto it.
   1. Relevance to Psychedelic Preparation: Psychedelic experiences often bring up challenging or intrusive thoughts. Learning to release distractions without resistance can help participants navigate intense moments with greater ease.
3. Relax (Pali: *Passambhayam* - पस्सम्भयं): Once a distraction is released, a subtle tension may still remain in the mind or body. The Relax step involves consciously softening any remaining tightness, both physically and mentally. This aligns with the Buddha’s instruction to “tranquilise” mental formations, allowing for a more effortless state of presence.
   1. Relevance to Psychedelic Preparation: Relaxation is key to reducing resistance and fear during a psychedelic journey. This step helps participants cultivate openness to whatever arises, preventing unnecessary struggle.
4. Re-Smile (Pali: *Pasannen* - पसन्नेन): The Re-Smile step encourages meditators to cultivate an attitude of lightness and ease. By gently smiling with the mind (and sometimes physically), practitioners reinforce a sense of joy and curiosity rather than frustration. This step ensures that the process remains one of engagement rather than struggle.
   1. Relevance to Psychedelic Preparation: Maintaining a light-hearted, accepting approach is particularly helpful when encountering challenging or unfamiliar experiences. The act of smiling naturally promotes positive neurochemical changes, reinforcing resilience and well-being.
5. Return (Pali: *Punarapi* - पुनरपि): After relaxing and re-smiling, the mind is gently returned to the object of meditation, whether it be the breath, a mantra, or the feeling of loving-kindness (*mettā*). This redirection is done effortlessly, without forcing focus.
   1. Relevance to Psychedelic Preparation: The ability to return to a stable anchor - such as the breath or the feeling of loving-kindness - helps participants navigate shifting states of consciousness during psychedelic experiences, providing a reliable point of reference.
6. Repeat (Pali: *Bahulikaritva* - बहुलीकरित्वा): The final step is to repeat the process as needed. Each time a distraction arises, the 6Rs cycle begins again, reinforcing the habit of mindful awareness, gentle letting go, and returning to presence.
   1. Relevance to Psychedelic Preparation: Repeating the 6Rs fosters habitual equanimity, allowing participants to develop the capacity to meet any experience - whether pleasurable or challenging - with stability and ease.

*Application of the 6Rs in the DIPP program*

The 6Rs method is progressively introduced and refined throughout the 21-day meditation training in DIPP (see **Fig. 2b** in the main text). It serves as the primary technique for managing distractions and sustaining a relaxed, open awareness, which are crucial skills for both meditation and psychedelic experiences.

- Early in the program (Days 1-7): Participants are introduced to the basic mechanics of the 6Rs and practice them in relation to cultivating loving-kindness for themselves.
- Middle phase (Days 8-14): They develop a more fluid and effortless application of the method, using it to sustain positive emotions without clinging.
- Later stages (Days 15-21): The 6Rs become an automatic process, integrated seamlessly into meditation practice, allowing awareness to rest in stillness without forced redirection.

By the end of the training, participants are deeply familiar with the 6Rs process, making it a valuable tool for navigating their upcoming psychedelic session with greater equanimity and ease.

For additional details on the 6Rs method, see [Dhamma Sukha Meditation Center](https://www.dhammasukha.org/).

**S2 The ‘Spectrum of Awareness’**

*Overview*

The Spectrum of Awareness is a structured model for understanding how awareness deepens and expands in meditation. It provides a practical framework for recognising different levels of experience, moving from sensory and cognitive content to more subtle states of awareness and eventually to open, formless awareness. This framework is a key component of the Digital Intervention for Psychedelic Preparation (DIPP) and supports participants in developing stability, insight, and equanimity as they navigate shifting states of consciousness.

Buddhist teachings emphasise direct awareness of experience rather than theoretical explanations. In this approach, as awareness deepens, we learn to observe experience with increasing clarity and spaciousness, leading to greater freedom from reactivity and suffering. The Spectrum of Awareness helps structure this deepening process.

*Phases of Awareness in the Spectrum*

Each phase represents a progressively more refined level of awareness, where prior phases remain present but become part of a broader experience.

1. Content (objects of awareness)
   1. The most concrete level of awareness focuses on objects of experience - thoughts, emotions, sensations, and perceptions.
   2. This is how awareness functions in daily life: distinguishing between a tree, a sound, a memory, or an emotion.
   3. Objects with high emotional charge (e.g., fear, desire) tend to dominate attention.
      1. Relevance to Psychedelic Preparation: During altered states, objects of awareness can become intensified. Training in recognising content helps participants engage with these experiences without becoming overwhelmed.
2. Processes (how awareness operates)
   1. As awareness relaxes and expands, we begin to see the mental processes that generate experience rather than just their content.
   2. Instead of focusing on a thought, we recognise the process of thinking; instead of fixating on an emotion, we see the process of feeling.
   3. This shift allows us to step back and observe without becoming entangled.
      1. Relevance to Psychedelic Preparation: Noticing mental processes rather than being consumed by them reduces reactivity to intense psychedelic phenomena.
3. Qualities of Awareness (the felt experience of awareness)
   1. Deeper awareness reveals the qualities that shape our perception - e.g., calmness, agitation, spaciousness, clarity.
   2. Awareness is no longer tied to specific objects or processes but feels into the underlying tone of experience.
   3. Recognising and adjusting these qualities cultivates balance and ease.
      1. Relevance to Psychedelic Preparation: Developing sensitivity to awareness qualities helps participants self-regulate emotions and responses during altered states.
4. Field of Awareness (awareness as a whole)
   1. At this stage, attention shifts to awareness itself rather than its contents or processes.
   2. Awareness is perceived as a spacious, all-encompassing field that includes everything.
   3. Instead of fixating on individual thoughts or emotions, we sense the entire field of experience.
      1. Relevance to Psychedelic Preparation: This perspective supports non-dual awareness and fosters a sense of unity and interconnectedness, which are common in psychedelic states.
5. Nothingness (open, formless awareness)
   1. In its most refined form, awareness becomes so expansive that it begins to dissolve into open nothingness.
   2. This phase manifests as gaps in perception - sometimes misinterpreted as micro-sleep but actually moments of cessation.
   3. In Buddhist teachings, this is known as nirodha (cessation) and is linked to profound states of insight and liberation.
      1. Relevance to Psychedelic Preparation: Encountering empty, formless awareness can be disorienting in altered states. Understanding this as a natural deepening of awareness helps participants navigate experiences of ego dissolution with equanimity.

*The Spectrum as a non-linear model*

While the Spectrum is often presented sequentially - from content to formless awareness - it is not a rigid, stepwise progression. Rather, these levels co-arise and influence each other.

- Awareness may shift dynamically between levels, especially in meditation or altered states.
- Some individuals may naturally experience higher levels first and later refine their ability to work with more concrete levels.
- Others may primarily engage with content and processes, gradually expanding into subtler levels over time.

Rather than treating the Spectrum as a hierarchical progression, it is best understood as a fluid, interactive system where all levels can be accessed and explored.

*Practical Applications in Meditation and Psychedelic Preparation*

The Spectrum of Awareness is integrated into DIPP training through guided meditation and reflective exercises. The practice involves:

1. Recognising where one’s awareness is currently anchored (content, process, qualities, field, or nothingness).
2. Gently shifting awareness along the Spectrum without force or expectation.
3. Using relaxation and expansion to facilitate movement across levels.
4. Developing familiarity with these shifts to navigate psychedelic experiences with greater stability and insight.

By incorporating the 6Rs method alongside the Spectrum of Awareness, participants learn to recognise distractions, release tension, and expand awareness in a structured, accessible way.

**S3 Informed consent form**

**
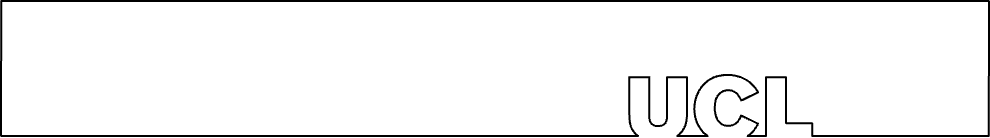
**

**Information Sheet for Healthy volunteers involved in a study using psilocybin**

**You will be given a copy of this information sheet.**

Title of Project: **‘Mechanisms of Tryptamines on Mental Health and Wellbeing’**

This study has been approved by the UCL Research Ethics Committee (Project ID Number): 19113/003

**Title of Study:** Mechanisms of Tryptamines on Mental Health and Wellbeing

Department: Experimental Psychology

UCL Division of Psychology and Language Sciences

University College London

26 Bedford Way

London

WC1H 0AP

**Name and Contact Details of the Researcher(s):**

Rosalind McAlpine: rosalind.mcalpine.18@ucl.ac.uk

Joanna Kuc: joanna.kuc.22@ucl.ac.uk

Magdalena Jaglinska: magdalena.jaglinska.22@ucl.ac.uk

Krisztina Jedlovszky: krisztina.jedlovszky.21@ucl.ac.uk

Ariel Castro: ariel.castro.24@ucl.ac.uk

**Name and Contact Details of the Principal Researchers:**

Sunjeev Kamboj, Email: sunjeev.kamboj@ucl.ac.uk

Jeremy Skipper, Email: Jeremy.skipper@ucl.ac.uk

Ravi Das, Email: xxxxxxx@xxxxxxx

**The purpose of this information sheet:**

We invite you to participate in a research project at UCL. There is no obligation to take part. You should *only* participate if you want to; choosing not to take part will not disadvantage you in any way. Before you decide, it is important for you to understand why the research is being done, and what participation will involve. Please take time to read the following information carefully and discuss it with others if you wish. Ask us if there is anything that is not clear or if you would like more information. Take time to decide whether or not you wish to take part. Thank you for reading this and considering participation in the study.

**What is the project’s purpose?**

Recent advancements in psychiatric research have highlighted the potential therapeutic benefits of psychedelic substances such as psilocybin. Our study, based at UCL, aims to explore the neural and subjective effects of psilocybin experiences, as well as any subsequent changes in psychological wellbeing and cognition.

In our study, you'll participate in a **specialised 21-day digital preparation program (‘DIPP’)** to prepare you for your psilocybin session, designed for easy completion at home. Upon enrollment, you'll gain access to a secure website with personalised login details, guiding you through daily and weekly preparatory exercises. This preparatory programme includes informative reading materials to build your understanding, reflective writing exercises to deepen your self-awareness, mood check-ins, and various other practices. These varied activities aim to mentally and emotionally equip you for a meaningful and safe psilocybin experience. Note: The course involves completing **up to 60 minutes of preparation every day, for 21 days**. **It is important to note that these preparation activities should ideally be completed in the morning, preferably as the first task of your day. Therefore, you should only sign up to participate if you can commit to morning sessions every day for the entire 21-day period.**

**Following the 21-day preparatory period, you will be invited to UCL for a session where you will be administered a dose of psilocybin, under the careful supervision of our experienced research team. Following this, we will monitor any long-term alterations in your mood, mental health and language for up to 9 months (see Section: ‘What will happen during testing?’ for more information).**

**Who are we recruiting?**

We would like to invite healthy males and females, aged 21-65. By ‘healthy’ we mean people who do not currently have a diagnosis and are not receiving any kind of treatment for a major mental or physical health condition. There may be some physical health conditions that are exceptions and we will need to discuss these with you during a telephone screen to check if you are eligible to take part.

Due to the nature of the drugs used in the study, we are also specifically looking for people with some prior experience of taking psychedelic drugs (e.g. LSD, psilocybin (magic mushrooms), DMT) but who have never used these drugs regularly or have an extensive history of use. If you have no experience with psychedelic drugs, you will not be able to take part. Similarly, if you have a history of mental health disorders or are taking medications you may not be able to take part. We have several other strict criteria, beyond those mentioned here, that we will need to check with you before being sure you are eligible to take part in the study. These criteria are in place to ensure your comfort and safety during the study, so it is very important that you meet them all.

**Do I have to take part?**

It is completely up to you to decide whether or not to take part. Please discuss this information sheet with friends, family members and your GP if you have any concerns. Please also feel free to discuss the information with the experimenters and ask any questions. If you do decide to take part you will be given this information sheet to keep and be asked to sign a consent form. You can withdraw from the study at any time without giving a reason. If you decide to withdraw we will not be able to use your data for the study and you will be asked what you wish to happen to the data you have provided up to that point. Once you have fully completed the study and received payment, you will no longer be able to withdraw your data from the study. The data will be fully anonymised at this point and you will not be identifiable from the data or any publications.

**Completing the pre-screening survey (what happens before you can take part)**

If after reading this sheet you decide you would still like to take part, you should complete the pre-screening survey. If you are eligible for participation at this stage, the research team will then organise a time to call you (via MS Teams) to run through a series of screening questions and check if you are eligible to take part. Please note that we have strict criteria for participation and based on your answers to these questions; you may not be eligible to take part in the study. If you are eligible to take part, you will be asked to come to the Clinical Psychopharmacology Unit (CPU) at UCL in central London at a time convenient for you.

The steps to take before you take part are:

1. Read this information sheet and complete the pre-screening survey (<https://www.psychedelicunit.com/dipp-prescreening>) if you think you might be eligible and would like to take part. If you do not wish to take part, there is no need to do anything, we will not collect or store any information about you.
2. Complete a telephone screening with the experimenter to assess if you are eligible. If you are not eligible, no information will be stored about you apart from your eligibility status.
3. Complete a clinical screening call with one of our clinical psychologists online.
4. Organise your UCL testing sessions with the experimenter.

**What will happen during testing?**

During your involvement in our study at UCL's Clinical Psychopharmacology Unit laboratories, you will undergo a series of structured events. Here's what you can expect:

*Visit 1: Baseline 1*

Once you meet our study's specific criteria and provide informed consent, we will enrol you into the study. You will be invited to UCL to complete a series of surveys, computer tasks, and provide a saliva sample for cortisol measurement (using the drool method). We will also record your heart activity with an electrocardiogram (ECG). We will also give you a wearable wristband (WHOOP), monitoring your heart-rate and temperature. We will ask you to wear the wristband everyday until Visit 4. This session will take around 3 hours.

During this visit, you will also be asked to confirm the name and contact details of the person who will collect you from UCL at the end of your dosing session on Visit 3. This designated person must be available and able to accompany you home safely after the session.

*Online Preparation Course (21 days)*

You will then be given access to our 21-day digital intervention platform (‘DIPP’) via a personalised login on our dedicated website. This platform will provide you with tasks designed to prepare you for the expected effects of the psychedelic substance, resources related to mental and physical preparedness, guidance on setting your intentions, recommendations for a safe psychedelic experience, and mood assessments. The course involves completing up to **60 minutes of preparation every day, for 21 days. It is important to note that these preparation activities should ideally be completed in the morning, preferably as the first task of your day. Therefore, you should only sign up to participate if you can commit to morning sessions every day for the entire 21-day period.**

You will also receive access to a **specialised mobile bot (‘DIPPBot’)** that will prompt you to provide voice note samples for 10 days leading up to your dosing and 10 days following it. The bot will be installed on your smartphone and will send you notifications twice a day, asking you to record and share a brief voice note about your inner thoughts.

*Visit 2: Pre-Dosing (-1 day)*

When you have completed your 21-day preparation programme, we will invite you to UCL to complete another round of surveys, computer tasks and undergo a brain scan while you watch a movie inside the fMRI scanner. We will also record the activity of your heart with ECG. This will take around 5 hours. You will have been provided with saliva collection kits at Visit 1. For Visit 2 you will need to collect a saliva sample between 1:00 PM and 4:00 PM. If your visit falls within this time frame, we will provide you with a new collection kit and ask you to provide the sample during your visit.

*Visit 3: Dosing Day*

The next day, you will have your psilocybin dosing. Your dosing session will take place in the labs at UCL and is expected to last **8-10 hours**. We ask you to fast in the morning, or have a light breakfast if needed, at least 2h before coming to UCL. Upon arrival, you will complete a short baseline questionnaire asking about your mood. We will provide you with a small drink of water to rinse your mouth, and you will be asked to provide a baseline saliva sample prior to dosing. A researcher will assist you with this using the swab method, where a small swab will be placed under your tongue, between your teeth, and allowed to rest for up to four minutes. This process is completely painless and minimally-invasive. After four minutes, the researcher will remove the swab from your mouth using personal protective equipment (PPE), such as gloves and forceps. This process will be completed at the end of each EEG scan, three times in total.

We will fit you with an EEG (electroencephalography) cap. This measures the electrical activity on your scalp produced by your brain. We will need to measure your head, fit a cap, and then fill the electrodes in the cap with a conductive gel. To get a good signal, we may need to gently rub your scalp to move hair out of the way of the electrodes. All of these procedures will be completely painless, but you will be left with conductive gel on your scalp, which you will be able to wash off in the testing centre. For this reason, we are unable to accept participants with hairstyles that would prevent us from attaching electrodes, or with a very small or large head circumference. You should not take part if you are uncomfortable having the cap fitted and gel applied to your scalp. The EEG cap will leave some slight red marks on your forehead where the electrodes press against the skin. These are not painful and will fade away within a few minutes of removing the cap. We will also place 3 ECG stickers on your right and left clavicle bones, and on your lower back to measure your heart rate. We will record EEG-ECG at baseline, 90 minutes and 150 minutes into your dosing session.

When you are comfortably settled, you will receive a capsule containing psilocybin. The room has been decorated to create a calming and pleasant atmosphere, and you will be provided with headphones to listen to a curated music playlist. Throughout the session, our team of experienced facilitators will be present to support you. They have been trained to guide the session in a non-directive manner, allowing you to explore your own experience without the constraints of a structured psychotherapy protocol. The facilitators will prioritise your safety and well-being, offering gentle guidance and reassurance when needed, while encouraging you to focus inward and engage with your unique psilocybin journey. You can trust that you will be in a secure, comfortable environment, surrounded by a compassionate and knowledgeable team dedicated to supporting you throughout the process.

After the effects of psilocybin wear off, our team will guide you through an 'integration' procedure to ensure you feel grounded. We'll ask you to share your experiences under the influence of psilocybin, and you'll be allowed to relax until you feel ready to leave. This integration process will be audio-recorded for subsequent analysis. Once you feel ready, there will be additional questionnaire measures and orientation tests to complete before you depart. Your designated person will collect you at the end of the day.

*Visit 4: Post-dosing (+2 weeks)*

Two weeks after dosing, we will invite you back to UCL to complete another round of surveys, computer tasks and an fMRI-movie brain scan. We will also record your heart activity with an ECG. This will take around **5 hours**. You will have been provided with a saliva collection kit at Visit 1. For Visit 4 you will need to collect a saliva sample between 1:00 PM and 4:00 PM. If your visit falls within this time frame, we will provide you with a new collection kit and ask you to provide the sample during your visit. The WHOOP wristband will be collected from you at this time and a researcher will assist you to delete the app from your phone.

*Online Follow-ups (1, 3, 6, 9 months)*

Following the study, we aim to monitor any long-term alterations in your mood, mental health, and language. At several intervals (1 month, 3 months, 6 months, 9 months), you'll be invited to fill out an online survey using a unique code provided to you. Filling out these questionnaires should take about 60 minutes of your time. These assessments will continue for up to 9 months after your dosing. Your participation in these follow-up surveys is vital, as it helps us gain insights into the lasting effects and potential benefits of the psychedelic experience.

**Drug and alcohol use during the study:**

Please abstain from using any recreational drugs during the duration of the study - this will be checked at the beginning each session. Moderate alcohol consumption and tobacco use are permitted between visits; however, you must avoid drinking alcohol for at least 24 hours before each in-person visit. Please note that smoking and vaping will not be allowed during Visit 3 (the psilocybin dosing day).

**What is psilocybin and is it safe?**

Psilocybin, the psychoactive compound found in certain species of mushrooms, has been the focus of recent therapeutic research. For participants in this study, it is important to understand that the psilocybin being used is sourced exclusively from Filament Health, an approved supplier accredited by the Medicines and Healthcare products Regulatory Agency (MHRA). This ensures that the compound provided is of the highest quality and safety, produced under stringent regulatory standards. By adhering to these rigorous guidelines, we can assure participants that the psilocybin utilised in our study is safe for clinical investigation and that its administration will be conducted under carefully controlled conditions, closely monitored by our team of healthcare professionals.

Psilocybin has been the subject of research in various clinical settings to assess its potential therapeutic effects, particularly in the context of mental health treatment. When administered in a controlled, clinical setting by experienced professionals, the use of psilocybin is generally considered to be safe, with a low risk of adverse reactions. It should be noted, however, that individual responses can vary, and participants may experience intense emotional and visual experiences. Some individuals may have adverse reactions, including anxiety and panic responses. In our study, we have implemented stringent safety protocols, including careful dosing and a supportive environment, to minimise any potential risks. Furthermore, participants will be under the supervision of trained healthcare professionals who are equipped to manage any adverse effects and ensure the safety and wellbeing of all participants during their psilocybin sessions. Prior to participating in the study, there will be a thorough screening process to identify any potential contraindications, helping to maintain a high level of safety throughout the study.

**Typical acute effects of psilocybin include:**

Shortly after consuming psilocybin, individuals often experience a range of acute effects, which can vary significantly between individuals. These effects generally last several hours (approx. 6). While some people find these experiences to be positive and potentially therapeutic, others may find them to be intense or unsettling. Typical acute effects of psilocybin may include:

- Changes in the perception of time and space
- Enhanced appreciation of colours and patterns
- Visual hallucinations (rarely auditory hallucinations)
- Feelings of euphoria, interconnectedness, or unity with the surroundings
- Sensations of transcendence or deep insight

Please note that individual experiences with psilocybin can be highly variable, and not all participants will experience all of the effects listed above. Our trained team will be available to support you throughout the experience to ensure your safety and wellbeing.

**What are the possible risks of taking part?**

While psilocybin is generally considered safe, it's important to understand that the experience induced by psilocybin can be overwhelming or emotionally challenging for some individuals. Following the session, some participants might feel a lingering sense of confusion or anxiety even after the drug's effects have subsided. Physical manifestations may include an elevated heart rate, increased blood pressure, feelings of restlessness, dizziness, rapid eye movements, and dilated pupils.

In individuals predisposed to certain mental health issues, particularly schizophrenia and psychosis, psilocybin could potentially exacerbate or trigger these conditions due to swift and intense shifts in brain chemistry. It's crucial that you refrain from participating if you or any close family members have a current or past history of these disorders.

Psilocybin interacts with the brain's serotonin receptors and, as such, might interfere with other drugs that influence serotonin levels, including antidepressants (SSRIs), monoamine oxidase inhibitors (MAOIs), amphetamines, and opioids. Participation is not permitted if you're currently on any of these medications.

Because of the profound subjective effects of psilocybin, we have a criterion that excludes participants without previous experience with psychedelics. We strive to minimise any challenging experiences both during and post the psilocybin session. To achieve this, we ensure you're well-prepared by discussing the process with seasoned members of our research team and providing clear information about psilocybin's safety. It's essential to remember that you're never obligated to partake and are free to exit the study at any point, even just before dosing. During the session, we offer a serene and comfortable setting, accompanied by music, which has shown to reduce the likelihood of negative experiences. After your session, there's ample opportunity to reflect on and discuss your experience with knowledgeable team members.

**What are the possible benefits of taking part?**

We hope that this work may in the future contribute to improving mental health therapies, elucidating the interplay between structured psychological preparation and psychedelic experiences. This encompasses its impacts on immediate neural responses (as gauged by EEG), subjective experiences during the psychedelic session, and prolonged alterations in language, cognition, and overall well being.

While we can't promise personal benefits such as elevated mood, many participants often find value in the tasks, fostering self-awareness about their cognitive abilities. Moreover, some individuals have reported enduring positive effects post-psychedelic sessions, ranging from profound self-insight to enhanced mood. However, it's imperative to note that individual experiences can vary, and we do make any claims that you will necessarily experience these benefits.

As a participant in this study, you will be compensated for your time and involvement at the standard UCL rate.. **You can expect to receive approximately £200 for your participation.** This payment will be processed after you complete Visit 4. If you have any questions or concerns regarding compensation, please contact the study coordinator. Please note that the final compensation amount may vary slightly depending on the exact duration of each visit.

**What if something goes wrong?**

If you have any questions about what happened/will happen during the study please contact one of the lead experimenter (Rosalind McAlpine: rosalind.mcalpine.18@ucl.ac.uk or Joanna Kuc: joanna.kuc.22@ucl.ac.uk). Should you wish to raise a complaint please contact the principal researcher (jeremy.skipper@ucl.ac.uk). We will do our very best to answer any questions and resolve any issues. If you feel your complaint cannot be handled to satisfaction through these routes you can contact the Chair of the UCL Research Ethics Committee – ethics@ucl.ac.uk.

**What do I do next if I want to take part?**

Once you have finished reading this information sheet, if you would still like to take part, you will need to complete an informed consent and screening process. This involves two steps; an online questionnaire, and a telephone call. The purpose of these questionnaires and calls is to make sure that you are eligible for the study, and to make sure that you understand exactly what your participation requires. As we are only accepting healthy volunteers for this trial, meaning no physical or mental health issues, we will have to ask you some questions about your mental health and drug use in the online questionnaire, and a few more over the phone. These questions relate to some potentially sensitive subjects which some people may find distressing to answer. Because of this, we’ll be asking for your consent to ask these questions and record your answers right at the start of the online screening questionnaire.

**Will my taking part in this project be kept confidential?**

Your participation in this research project will be treated with utmost confidentiality. All information collected will be pseudonymized, meaning that it will be linked to you only via a unique identifier known solely to the research team. This data will be securely stored on encrypted university servers until analysis is complete. It will then be retained only until it is no longer required for research purposes, at which point it will be destroyed along with the linkage code. Thereafter, data will be fully anonymized and may be shared with other researchers or repositories for future scientific studies, ensuring that it cannot be traced back to you. In the event that data needs to be stored indefinitely for ongoing research, it will be held in an anonymized format in a secure environment, with access strictly controlled according to data protection regulations. Our commitment to maintaining the confidentiality of your data adheres to the highest standards of data management and ethical research guidelines.

**Participant data withdrawal**

As a participant, you are entitled to withdraw your personal data from this research project up to 60 days following your dosing day. During the course of the study and this 30-day period, your identifiable information will be securely stored on a Windows-encrypted spreadsheet. The retention of your identifiable information during this time is essential, as it enables us to respond to any withdrawal requests by providing you with all the data collected on you and removing it from the study records if you choose to withdraw. After this time, on a specified date, your data will be anonymized, meaning all personal identifiers will be permanently removed. Once anonymization has occurred, it will no longer be possible to extract or delete individual participant data. We encourage you to make any decisions regarding the withdrawal of your data promptly and to communicate them to us well before the 60-day post-dosing deadline. This ensures your choices regarding your data are fully honoured and executed in accordance with your wishes.

**What will happen to the results of the research project?**

The results of the study will be published in scientific peer-reviewed journals. You will not be identifiable from the data or any publications. If you would like to receive an overview of the study’s results once it has been completed, please ask the investigator and this will be arranged.

**Local Data Protection Privacy Notice:**

The controller for this project will be University College London (UCL). The UCL Data Protection Officer provides oversight of UCL activities involving the processing of personal data, and can be contacted at data-protection@ucl.ac.uk

This ‘local’ privacy notice sets out the information that applies to this particular study. Further information on how UCL uses participant information can be found in our ‘general’ privacy notice. For participants in research studies, click [here](https://www.ucl.ac.uk/legal-services/privacy/ucl-general-research-participant-privacy-notice)

The information that is required to be provided to participants under data protection legislation (GDPR and DPA 2018) is provided across both the ‘local’ and ‘general’ privacy notices.

The categories of personal data used will be as follows:

● Name

● Work Phone number

● Email address

● Date of Birth

The lawful basis that would be used to process your *personal data* will be performance of a task in the public interest.

The lawful basis used to process *special category personal data* will be for scientific and historical research or statistical purposes.

*Your personal data will be processed so long as it is required for the research project*. If we are able to anonymise or pseudonymise the personal data you provide we will undertake this, and will endeavour to minimise the processing of personal data wherever possible.

If you are concerned about how your personal data is being processed, or if you would like to contact us about your rights, please contact UCL in the first instance at data-protection@ucl.ac.uk.

**Contact for further information**

**Name and Contact Details of the Researcher:**

Rosalind McAlpine: rosalind.mcalpine.18@ucl.ac.uk

Joanna Kuc: joanna.kuc.22@ucl.ac.uk

Magdalena Jaglinska: magdalena.jaglinska.22@ucl.ac.uk

Krisztina Jedlovszky: krisztina.jedlovszky.21@ucl.ac.uk

Ariel Castro: ariel.castro.24@ucl.ac.uk

**Name and Contact Details of the Principal Researchers:**

Sunjeev Kamboj, Email: sunjeev.kamboj@ucl.ac.uk

Dr. Jeremy Skipper, email: Jeremy.skipper@ucl.ac.uk

Dr Ravi Das, Email: xxxxxxxx@xxxxxx

**Thank you for reading this information sheet and for considering taking part in this research study.**

**—-----------------------------------------------------------------------------------------------------------------------------------------**

## **CONSENT FORM FOR PARTICIPANTS INVOLVED IN RESEARCH STUDIES USING PSILOCYBIN**

**Please complete this form after you have read the Information Sheet and/or listened to an explanation about the research.**

**Title of Study:** Mechanisms of Tryptamines on Mental Health and Wellbeing

**Department:** Clinical, Educational and Health Psychology, Psychology and Language Sciences Name and

**Contact Details of the Researcher(s):**

Rosalind McAlpine: rosalind.mcalpine.18@ucl.ac.uk

Joanna Kuc: joanna.kuc.22@ucl.ac.uk

Magdalena Jaglinska: magdalena.jaglinska.22@ucl.ac.uk

Krisztina Jedlovszky: krisztina.jedlovszky.21@ucl.ac.uk

Ariel Castro: ariel.castro.24@ucl.ac.uk

**Name and Contact Details of the Principal Researcher:**

Sunjeev Kamboj, Email: sunjeev.kamboj@ucl.ac.uk

Dr Jeremy Skipper (jeremy.skipper@ucl.ac.uk).

**Name and Contact Details of the UCL Data Protection Officer:**

Alex Potts. Email xxxxxx@xxxxxx

If you are concerned about how your personal data is being processed, or if you would like to contact someone about your rights, you can contact UCL at data-protection@ucl.ac.uk. This study has been approved by the UCL Research Ethics Committee: Project ID number: 19113/003.

- - - - - -

Thank you for considering taking part in this research. The person organising the research must explain the project to you before you agree to take part. If you have any questions arising from the Information Sheet or explanation already given to you, please ask the researcher before you decide whether to join in. You will be given a copy of this Consent Form to keep and refer to at any time.

**I understand that by marking each box below, I am giving my consent to participate in that specific aspect of the study. If a box remains unmarked, it indicates that I do not give my consent to participate in that portion of the study. Please be aware that not consenting to certain elements may mean I am not eligible to participate in the study.**

| **Statement** | **Tick** |
| --- | --- |
| I have read and understood the Information Sheet for the above study, had the opportunity to consider the information, ask questions, and have received satisfactory answers. |  |
| I understand that I can withdraw my data up to the end of the dosing visit. |  |
| I consent to participate in the study under the conditions explained regarding data protection and use. |  |
| I understand that all personal information will remain confidential to the extent explained, with some potential exceptions for legal and safety reasons. |  |
| I understand that individuals from the University, sponsors, and funders might review my information for monitoring and audit purposes. |  |
| I understand my participation is voluntary and I can withdraw at any time without giving a reason, with the option to have my data deleted. |  |
| I am aware of the potential risks and the available support if I become distressed during the research. |  |
| I understand the direct and indirect benefits of participating. |  |
| I understand that data from this study will not be shared with commercial organisations and is the responsibility of the research team. |  |
| I consent to have my interview audio recorded under the specified conditions. |  |
| I understand the inclusion criteria as detailed and explained by the researcher. |  |
| I have disclosed any potential reasons, including health and coercion concerns, that might prevent my participation in the study. |  |
| I confirm that I am not pregnant, breastfeeding, or planning to become pregnant during the study. |  |
| I agree to abstain from illicit drug use from the start of the preparation course to one week post dosing. |  |
| I voluntarily agree to participate in this study. |  |

**By ticking each box, you are confirming your willingness to participate in each integral component of the study. Please review each participation aspect carefully and indicate your agreement by ticking the corresponding box:**

| **Participation Aspect** | **Tick** |
| --- | --- |
| Participation in an individual screening interview |  |
| Participant in fMRI-movie scans during Visit 1 and Visit 2 |  |
| Completion of the 21-day online preparation course |  |
| Attendance and participation in the psilocybin dosing session at UCL |  |
| Undergoing EEG recordings to capture brain activity |  |
| Undergoing ECG recordings to capture heart activity |  |
| Completing psychological questionnaires, which may involve discussing sensitive topics |  |
| Completing cognitive tasks on a computer |  |
| Contributing to the remote sampling of inner experience via regular voice recordings |  |
| Wearing WHOOP wristbands monitoring real-life physiological signals, including heart rate |  |
| Providing salivary samples for cortisol measurement |  |

**If you would like your contact details to be retained so that you can be contacted in the future by UCL researchers who would like to invite you to participate in follow up studies to this project, or in future studies of a similar nature, please tick the appropriate box below.**

| **Yes, I would be happy to be contacted in this way** |  |
| --- | --- |
| **No, I would not like to be contacted** |  |

_________________ ____________________ _______________________

Participant name Date Signature

_________________ ____________________ _______________________

Researcher name Date Signature

**S4 Study timeline and outcome measures**

**SPIRIT table**

|  | **STUDY PERIOD** | | | | | | | |
| --- | --- | --- | --- | --- | --- | --- | --- | --- |
|  | **Online** | **On-site** | | | | **Online** | | |
| **TIMEPOINT** | **-t_1_** | **t_1_** | **t_2_** | **t_3_** | **t_4_** | **t_5_** | **t_6_** | **t_7_** |
| **ENROLMENT:** | | | | | | | | |
| Online screening | X |  |  |  |  |  |  |  |
| Telephone screening | X |  |  |  |  |  |  |  |
| Clinical screening | X |  |  |  |  |  |  |  |
| Informed consent | X |  |  |  |  |  |  |  |
| Allocation | X |  |  |  |  |  |  |  |
| **INTERVENTIONS:** | | | | | | | | |
| DIPP |  |  | |  |  |  |  |  |
| DIPP-NM |  |  | |  |  |  |  |  |
| Psilocybin (25 mg) |  |  |  | X |  |  |  |  |
| **PRIMARY OUTCOMES:** | | | | | | | | |
| Recruitment efficiency | X |  |  |  |  |  |  |  |
| Study retention |  | | | | |  |  |  |
| DIPP intervention adherence |  |  | |  |  |  |  |  |
| **SECONDARY OUTCOMES:** | | | | | | | | |
| Subjective Feasibility of Intervention Scale (SFIS) |  |  | X |  |  |  |  |  |
| Theoretical Framework of Acceptability Scale (TFA) |  |  | X |  |  |  |  |  |
| System Usability Scale (SUS) |  |  | X |  |  |  |  |  |
| Mobile Application Rating Scale (MARS) |  |  | X |  |  |  |  |  |
| Psychedelic Preparedness Scale (PPS) |  | X | X |  |  |  |  |  |
| Altered States Consciousness Questionnaire (ASC) |  |  |  | X |  |  |  |  |
| Challenging Experience Questionnaire (CEQ) |  |  |  | X |  |  |  |  |
| Short Warwick-Edinburgh Mental Wellbeing Scale (SWEMWBS) |  | X | X | X | X | X | X | X |
| **OTHER PRE-SPECIFIED OUTCOMES:** | | | | | | | | |
| Credibility and Expectations Questionnaire (CEQ) |  | X |  |  |  |  |  |  |
| DIPP-based mood and meta-emotional awareness tracking |  | X | X |  |  |  |  |  |
| DIPP-Bot inner speech metrics |  | X | X |  |  |  |  |  |
| Heart Rate Variability (ECG) |  | X | X | X | X |  |  |  |
| Neural activity (EEG) |  |  |  | X |  |  |  |  |
| Neural activity (movie-fMRI) |  |  | X |  | X |  |  |  |
| Computerised Restless 3-Arm Bandit Task |  | X | X |  | X |  |  |  |
| Computerised Body Maps of Emotion Tool (emBODY) |  | X | X |  | X |  |  |  |
| Computerised Perceptual Reality Monitoring Task |  | X |  |  | X |  |  |  |
| Cognitive and Affective Mindfulness Scale - Revised (CAMS-R) |  | X | X |  | X | X | X | X |
| Sussex-Oxford Compassion for the Self Scale (SOCS-S) |  | X | X |  | X | X | X | X |
| Open and Engaged State Questionnaire (OESQ) |  | X | X |  | X | X | X | X |
| Patient Health Questionnaire 9 (PHQ-9) |  | X | X |  | X | X | X | X |
| Generalised Anxiety Disorder 7 (GAD-7) |  | X | X |  | X | X | X | X |
| Ruminative Response Scale - Short form (RRS-SF) |  | X | X |  | X | X | X | X |
| Varieties of Inner Speech Questionnaire - Revised (VISQ-R) |  | X | X |  | X | X | X | X |
| Nevada Inner Experience Questionnaire (NIEQ) |  | X | X |  | X | X | X | X |
| Perseverative Thinking Questionnaire (PTQ) |  | X | X |  | X | X | X | X |
| Brief Multidimensional Assessment of Interoceptive Awareness, version 2 (Brief MAIA-2) |  | X | X |  | X | X | X | X |
| Černis Felt Sense of Anomaly Scale - Short form (ČEFSA) |  | X | X |  | X | X | X | X |
| 9-item Material Values Scale (MVS) |  | X |  |  | X | X | X | X |
| Emotional Breakthrough Inventory (EBI) |  |  |  | X |  |  |  |  |
| Geneva Emotional Music Scale - 9 (GEMS-9) |  |  |  | X |  |  |  |  |
| Psychedelic Music Questionnaire - Short Form (PMQ-SF) |  |  |  | X |  |  |  |  |
| Positive and Negative Affect Schedule (PANAS) |  |  |  | X |  |  |  |  |
| Drug Effects Questionnaire (DEQ) |  |  |  | X |  |  |  |  |
| 6-item Dissociative Symptom Scale (CADSS-6) |  |  |  | X |  |  |  |  |
| Saliva samples for cortisol analysis |  | X | X | X | X |  |  |  |
| WHOOP wearable devices for continuous biometric tracking |  |  | | | |  |  |  |

**SPIRIT checklist**

| **Section / Topic** | **No** | **SPIRIT 2025 checklist item description** | **Reported on page no.** |
| --- | --- | --- | --- |
| Administrative information | | |  |
| Title and structured summary | 1a | Title stating the trial design, population, and interventions, with identification as a protocol | 1 |
|  | 1b | Structured summary of trial design and methods, including items from the World Health Organization Trial Registration Data Set | 11,12 |
| Protocol version | 2 | Version date and identifier | 1 |
| Roles and responsibilities | 3a | Names, affiliations, and roles of protocol contributors | 1 |
|  | 3b | Name and contact information for the trial sponsor | 18 |
|  | 3c | Role of trial sponsor and funders in design, conduct, analysis, and reporting of trial; including any authority over these activities | 18 |
|  | 3d | Composition, roles, and responsibilities of the coordinating site, steering committee, endpoint adjudication committee, data management team, and other individuals or groups overseeing the trial, if applicable | 17 |
| Open science | | |  |
| Trial registration | 4 | Name of trial registry, identifying number (with URL), and date of registration. If not yet registered, name of intended registry | 2 |
| Protocol and statistical analysis plan | 5 | Where the trial protocol and statistical analysis plan can be accessed | 13 |
| Data sharing | 6 | Where and how the individual de-identified participant data (including data dictionary), statistical code, and any other materials will be accessible | 17 |
| Funding and conflicts of interest | 7a | Sources of funding and other support (e.g., supply of drugs) | 17 |
|  | 7b | Financial and other conflicts of interest for principal investigators and steering committee members | 17 |
| Dissemination policy | 8 | Plans to communicate trial results to participants, healthcare professionals, the public, and other relevant groups (e.g., reporting in trial registry, plain language summary, publication) | 16 |
| Introduction | | |  |
| Background and rationale | 9a | Scientific background and rationale, including summary of relevant studies (published and unpublished) examining benefits and harms for each intervention | 4 |
|  | 9b | Explanation for choice of comparator | 8 |
| Objectives | 10 | Specific objectives related to benefits and harms | 12 |
| Methods: Patient and public involvement, trial design | | |  |
| Patient and public involvement | 11 | Details of, or plans for, patient or public involvement in the design, conduct, and reporting of the trial | 8 |
| Trial design | 12 | Description of trial design including type of trial (e.g., parallel group, crossover), allocation ratio, and framework (e.g., superiority, equivalence, non-inferiority, exploratory) | 11 |
| Methods: Participants, interventions, and outcomes | | |  |
| Trial setting | 13 | Settings (e.g., community, hospital) and locations (e.g., countries, sites) where the trial will be conducted | 8 |
| Eligibility criteria | 14a | Eligibility criteria for participants | 9 |
|  | 14b | If applicable, eligibility criteria for sites and for individuals who will deliver the interventions (e.g., surgeons, physiotherapists) | 15 |
| Intervention and comparator | 15a | Intervention and comparator with sufficient details to allow replication including how, when, and by whom they will be administered. If relevant, where additional materials describing the intervention and comparator (e.g., intervention manual) can be accessed | 5 |
|  | 15b | Criteria for discontinuing or modifying allocated intervention/comparator for a trial participant (e.g., drug dose change in response to harms, participant request, or improving/worsening disease) | 17 |
|  | 15c | Strategies to improve adherence to intervention/comparator protocols, if applicable, and any procedures for monitoring adherence (e.g., drug tablet return, sessions attended) | 12 |
|  | 15d | Concomitant care that is permitted or prohibited during the trial | 9 |
| Outcomes | 16 | Primary and secondary outcomes, including the specific measurement variable (e.g., systolic blood pressure), analysis metric (e.g., change from baseline, final value, time to event), method of aggregation (e.g., median, proportion), and time point for each outcome | 12 |
| Harms | 17 | How harms are defined and will be assessed (e.g., systematically, non-systematically) | 14 |
| Participant timeline | 18 | Time schedule of enrollment, interventions (including any run-ins and washouts), assessments, and visits for participants. A schematic diagram is highly recommended (see Figure) | 13 (S4) |
| Sample size | 19 | How sample size was determined, including all assumptions supporting the sample size calculation | 9 |
| Recruitment | 20 | Strategies for achieving adequate participant enrollment to reach target sample size | 9 |
| Methods: Assignment of interventions | | |  |
| Randomization: |  |  |  |
| Sequence generation | 21a | Who will generate the random allocation sequence and the method used | 11 |
|  | 21b | Type of randomization (simple or restricted) and details of any factors for stratification. To reduce predictability of a random sequence, other details of any planned restriction (e.g., blocking) should be provided in a separate document that is unavailable to those who enroll participants or assign interventions | 11 |
| Allocation concealment  mechanism | 22 | Mechanism used to implement the random allocation sequence (e.g., central computer/telephone; sequentially numbered, opaque, sealed containers), describing any steps to conceal the sequence until interventions are assigned | 11 |
| Implementation | 23 | Whether the personnel who will enroll and those who will assign participants to the interventions will have access to the random allocation sequence | 11 |
| Blinding | 24a | Who will be blinded after assignment to interventions (e.g., participants, care providers, outcome assessors, data analysts) | 11 |
|  | 24b | If blinded, how blinding will be achieved and description of the similarity of interventions | 11 |
|  | 24c | If blinded, circumstances under which unblinding is permissible, and procedure for revealing a participant’s allocated intervention during the trial | 11 |
| Methods: Data collection, management, and analysis | | |  |
| Data collection methods | 25a | Plans for assessment and collection of trial data, including any related processes to promote data quality (e.g., duplicate measurements, training of assessors) and a description of trial instruments (e.g., questionnaires, laboratory tests) along with their reliability and validity, if known. Reference to where data collection forms can be accessed, if not in the protocol | 12 (S5) |
|  | 25b | Plans to promote participant retention and complete follow-up, including list of any outcome data to be collected for participants who discontinue or deviate from intervention protocols | 12 |
| Data management | 26 | Plans for data entry, coding, security, and storage, including any related processes to promote data quality (e.g., double data entry; range checks for data values). Reference to where details of data management procedures can be accessed, if not in the protocol | 14 |
| Statistical methods | 27a | Statistical methods used to compare groups for primary and secondary outcomes, including harms | 13 |
|  | 27b | Definition of who will be included in each analysis (e.g., all randomized participants), and in which group | 13 |
|  | 27c | How missing data will be handled in the analysis | 13 |
|  | 27d | Methods for any additional analyses (e.g., subgroup and sensitivity analyses) | 13 |
| Methods: Monitoring | | |  |
| Data monitoring committee | 28a | Composition of data monitoring committee (DMC); summary of its role and reporting structure; statement of whether it is independent from the sponsor and funder; conflicts of interest and reference to where further details about its charter can be found, if not in the protocol. Alternatively, an explanation of why a DMC is not needed | 14 |
|  | 28b | Explanation of any interim analyses and stopping guidelines, including who will have access to these interim results and make the final decision to terminate the trial | 14 |
| Trial monitoring | 29 | Frequency and procedures for monitoring trial conduct. If there is no monitoring, give explanation | 14 |
| Ethics | | |  |
| Research ethics approval | 30 | Plans for seeking research ethics committee/institutional review board approval | 17 |
| Protocol amendments | 31 | Plans for communicating important protocol modifications to relevant parties | 17 |
| Consent or assent | 32a | Who will obtain informed consent or assent from potential trial participants or authorized proxies, and how |  |
|  | 32b | Additional consent provisions for collection and use of participant data and biological specimens in ancillary studies, if applicable | 17 |
| Confidentiality | 33 | How personal information about potential and enrolled participants will be collected, shared, and maintained in order to protect confidentiality before, during, and after the trial | 17 |
| Ancillary and post-trial care | 34 | Provisions, if any, for ancillary and post-trial care, and for compensation to those who suffer harm from trial participation | 17 |

| **S5 Primary, S5 Primary, secondary and other pre-specified outcomes** | | |
| --- | --- | --- |
| **OUTCOME MEASURE** | **DESCRIPTION** | **TIMEFRAME** |
| **PRIMARY** | | |
| Recruitment efficiency | Weekly rate of participant enrollment and randomisation. Success is defined as ≥1 participant per week (average) until target sample (N=40) achieved. | -t_1_ (until end of recruitment period) |
| Study retention | Percentage of randomised participants completing the 2-week post-dose follow-up assessment. Success is defined as ≥70% completion rate. | t_1_,t_4_ |
| DIPP intervention adherence | Task completion rates across three daily tasks (meditation/music practice, mood rating, journal entry) and two weekly tasks. Success is defined as ≥70% of participants achieving an average completion rate of ≥70% across all required tasks. | t_1_,t_2_ |
| **SECONDARY** | | |
| DIPP platform feasibility as measured by the Subjective Feasibility of Intervention Scale (SFIS) | The Subjective Feasibility of Intervention Scale (SFIS) measures perceived practicality, resource demands and integration into existing workflows of the DIPP protocol. Possible scores range from 9 to 45, with higher scores indicating better feasibility. | t_2_ |
| DIPP platform acceptability as measured by the Theoretical Framework of Acceptability Scale (TFA) [(1)](https://paperpile.com/c/0oMQDJ/7oqrq) | The Theoretical Framework of Acceptability Scale (TFA) measures perceived appropriateness and satisfaction with the DIPP platform. Possible scores range from 7 to 35, with higher scores indicating better acceptability. | t_2_ |
| DIPP platform usability and engagement as measured by the System Usability Scale (SUS) [(2)](https://paperpile.com/c/0oMQDJ/mHb9y) and Mobile Application Rating Scale (MARS) [(3)](https://paperpile.com/c/0oMQDJ/38YS7) | The System Usability Scale (SUS) and Mobile Application Rating Scale (MARS; functionality and aesthetics subscales only) measure platform user-friendliness and app quality respectively. Combined scores range from 16 to 80, with higher scores indicating a better usability/engagement. | t_2_ |
| Psychedelic Preparedness as measured using the Psychedelic Preparedness Scale (PPS) [(4)](https://paperpile.com/c/0oMQDJ/0CZdo) | Between-group differences in psychedelic preparedness will be assessed using the Psychedelic Preparedness Scale (PPS), a validated 20-item self-report measure. The PPS evaluates four domains: knowledge-expectation, psychophysical-readiness, intention-preparation, and support-planning. Individual total scores (range: 20-140) and mean factor scores (range: 1-7) will be calculated, with higher scores indicating greater preparedness. | t_1_,t_2_ |
| Positive quality of acute psychedelic experience as measured by the OBN dimension from the Altered States Consciousness Questionnaire (ASC) [(5)](https://paperpile.com/c/0oMQDJ/LP6wn) | The Altered States Consciousness Questionnaire (11D-ASC) measures the subjective effects of the psychedelic experience. To assess the positive quality of the experience, this study will focus on the Oceanic Boundlessness (OBN) factor to assess participants’ subjective experience of unity, boundary dissolution, and interconnectedness during the psilocybin session. Possible standardised scores range from 0 to 1, with higher scores indicating a more positive quality of acute psychedelic experience. | t_3_ |
| Challenging psychological experience during the psychedelic experience as measured by four subscales from the Challenging Experience Questionnaire (CEQ) [(6)](https://paperpile.com/c/0oMQDJ/nXd62) | The Challenging Experience Questionnaire (CEQ) measures various aspects of challenge experienced during a psychedelic experience. To assess challenging psychological experiences during the psilocybin session, this study will focus on the combined mean score on four CEQ subscales (Fear, Insanity, Isolation, and Paranoia). Possible scores range from 0 to 5, with higher scores indicating a more challenging psychedelic experience. | t_3_ |
| Mental wellbeing as measured by the Warwick-Edinburgh Mental Wellbeing Scale (WEMWBS) [(7)](https://paperpile.com/c/0oMQDJ/WZly9) | The Warwick-Edinburgh Mental Wellbeing Scale (WEMWBS) measures psychological functioning and emotional wellbeing. Possible scores range from 14 to 70, with higher scores indicating better wellbeing. | t_1_,t_2_,t_3_,t_4_ |
| **OTHER PRE-SPECIFIED** | | |
| Credibility and Expectations Questionnaire-D (CEQ-D) [(8)](https://paperpile.com/c/0oMQDJ/S6zd) | The CEQ-D is an adapted version of the Credibility and Expectations Questionnaire designed to assess participants’ perceived credibility and anticipated effectiveness of the DIPP intervention for psychedelic preparedness. The measure comprises six items divided across two subscales: cognitive credibility (e.g., logical coherence, anticipated usefulness, willingness to recommend) and affective expectancy (e.g., intuitive belief in benefit). Responses are rated on 9-point Likert scales or 10-point percentage scales, with higher scores indicating stronger belief in the intervention’s plausibility and potential impact | t_1_ |
| DIPP-based mood and meta-emotional awareness tracking | Participants will complete a daily mood check-in after each session during the 21-day DIPP, which includes (1) five questions on state mindfulness, (2) two on decentering, (3) identifying their emotion on a valence-arousal grid, and (4) rating the accuracy of their emotion identification (meta-emotional awareness). | t_1_,t_2_ |
| DIPPBot inner speech metrics | The DIPP-Bot mobile app captures daily voice note samples to assess changes in spontaneous inner speech patterns. The analysis will encompass linguistic, syntactic, and acoustic domains, leveraging advanced Natural Language Processing (NLP) techniques and sound feature analysis to evaluate measurable changes in the content, structure, and vocal characteristics of participants’ inner dialogue over time. | t_1_,t_2_ |
| Physiological activity as measured by Electrocardiography (ECG) | Electrocardiography (ECG) measures the electrical activity of the heart. A three-lead eyes-closed ECG will be used to record cardiac activity before, during, and after the intervention. During the peak effects of psilocybin, ECG will be recorded for 7 minutes at 90 and 150 minutes after ingestion of the drug. The analysis will evaluate autonomic nervous system function based on the Heart Rate Variability (HRV) metrics. | t_1_,t_2_,t_3_,t_4_ |
| Neural activity as measured by Electroencephalography (EEG) | Electroencephalography (EEG) measures brain electrical activity during the psychedelic experience. Eyes-closed EEG will be recorded at baseline and at 90 and 150 minutes after ingestion of psilocybin. The analysis will evaluate spectral power across frequency bands, neural complexity, connectivity, and traveling wave dynamics. Additionally, Heartbeat-Evoked Potentials (HEP) will be analysed to assess changes in cortical processing of cardiac interoceptive signals. | t_3_ |
| Neural activity (movie-fMRI) | Functional Magnetic Resonance Imaging (fMRI) data will be collected while participants watch full length feature movies at baseline and 2-week follow-up. The analysis will assess lasting changes in brain networks following psilocybin administration, and whether these changes are associated with well-being outcomes. | t_2_,t_4_ |
| Computerised Restless 3-arm Bandit Task [adapted from Ramaswamy et al., *(in prep*)] | The restless 3-arm bandit task asks participants to make decisions selecting different boxes (arms) that yield rewards and punishments with changing probabilities. The task provides a range of computational measures that capture clinically relevant aspects of reward sensitivity and reward seeking. Possible measures include reward learning rate, exploration rate, reward seeking and loss aversion. | t_1_,t_2_,t_4_ |
| Computerised Body Maps of emotions Task Computerised Body Maps of Emotions Tool (emBODY) [(9)](https://paperpile.com/c/0oMQDJ/1AFRQ) | The emBODY tool is a computerised task used for mapping emotion-dependent sensations in distinct body regions. This study uses an online version adapted from the original task. Analysis includes changes in strength of bodily sensations linked to specific emotions and emotional granularity, measured as the spatial differentiation of emotional experiences. | t1,t2,t4 |
| Computerised perceptual reality monitoring task [adapted from [(10)](https://paperpile.com/c/0oMQDJ/TJxMR)] | The perceptual reality monitoring task consists of participants imagining and making judgements about a set of Gabor gratings. The task yields psychophysical measures about the sensitivity of perception and mental imagery, as well as confidence in perceptual judgements. | t_1_,t_2_,t_4_ |
| Mindfulness capacity as measured by the Cognitive and Affective Mindfulness Scale Revised (CAMS-R) [(11)](https://paperpile.com/c/0oMQDJ/dDojl) | The Cognitive and Affective Mindfulness Scale Revised (CAMS-R) measures mindfulness capacity across attention regulation, emotional tolerance, and present-moment awareness. Possible scores range from 12 to 48, with higher scores indicating a better outcome. | t_1_,t_2_,t_3_,t_4,_t_5_,t_6_,t_7_ |
| Self-compassion as measured by the Sussex-Oxford Compassion for the Self Scale (SOCS-S) [(12)](https://paperpile.com/c/0oMQDJ/kg37z) | The Sussex-Oxford Compassion for the Self Scale (SOCS-S) measures self-compassion across recognizing suffering, understanding universality, feeling compassion, tolerating uncomfortable emotions, and motivation to alleviate suffering. Possible scores range from 20 to 100, with higher scores indicating a better outcome. | t_1_,t_2_,t_3_,t_4,_t_5_,t_6_,t_7_ |
| Psychological flexibility as measured by the Open and Engaged State Questionnaire (OESQ) [(13)](https://paperpile.com/c/0oMQDJ/erhKC) | The Open and Engaged State Questionnaire (OESQ) measures openness and engagement with emotions and life circumstances. Possible scores range from 0 to 40, with higher scores indicating a better outcome. | t_1_,t_2_,t_3_,t_4,_t_5_,t_6_,t_7_ |
| Depression severity as measured by the Patient Health Questionnaire (PHQ-9) [(14)](https://paperpile.com/c/0oMQDJ/cNV9M) | The Patient Health Questionnaire (PHQ-9) measures depressive symptoms, including mood, sleep, energy levels, appetite, and suicidal thoughts. Possible scores range from 0 to 27, with higher scores indicating a worse outcome. | t_1_,t_2_,t_3_,t_4,_t_5_,t_6_,t_7_ |
| Anxiety severity as measured by the Generalised Anxiety Disorder 7 (GAD-7) [(15)](https://paperpile.com/c/0oMQDJ/GQccF) | The Generalised Anxiety Disorder (GAD-7) measures anxiety symptoms, including worry, restlessness, irritability, and physical symptoms. Possible scores range from 0 to 21, with higher scores indicating a worse outcome. | t_1_,t_2_,t_3_,t_4,_t_5_,t_6_,t_7_ |
| Rumination as measured by the Ruminative Response Scale (RRS-SF) [(16)](https://paperpile.com/c/0oMQDJ/MjFG) | The Short Form Ruminative Response Scale (RRS-SF10) measures the tendency to have persistent negative thinking patterns during periods of low mood. Possible scores range from 10 to 40, with higher scores indicating a higher tendency to ruminate. | t_1_,t_2_,t_3_,t_4,_t_5_,t_6_,t_7_ |
| Varieties of Inner Speech Questionnaire - Revised (VISQ-R) [(17)](https://paperpile.com/c/0oMQDJ/KIW8) | The Varieties of Inner Speech Questionnaire - Revised (VISQ-R) measures different forms of inner speech, including dialogical inner speech, condensed inner speech, other voices in inner speech, evaluative/critical inner speech, and positive/regulatory inner speech. Possible scores in each dimension range from 1 to 7, with higher scores indicating a greater frequency or prominence of that type of inner speech. | t_1_,t_2_,t_3_,t_4,_t_5_,t_6_,t_7_ |
| Inner experience types as measured by the Nevada Inner Experience Questionnaire (NIEQ) [(18)](https://paperpile.com/c/0oMQDJ/gfVh) | The Nevada Inner Experience Questionnaire (NIEQ) measures various types of inner experiences, including inner speech, visual imagery, emotional experience, sensory awareness, and non-symbolic thought. Possible scores in each dimension range from 5 to 50, with higher scores indicating a greater frequency or prominence of that type of inner experience. | t_1_,t_2_,t_3_,t_4,_t_5_,t_6_,t_7_ |
| Perseverative thinking as measured by the Perseverative Thinking Questionnaire (PTQ) [(19)](https://paperpile.com/c/0oMQDJ/tXbLx) | The Perseverative Thinking Questionnaire (PTQ) measures repetitive negative thinking as a response to negative experiences. Possible scores range from 0 to 60, with higher scores indicating a higher tendency for repetitive negative thinking. | t_1_,t_2_,t_3_,t_4,_t_5_,t_6_,t_7_ |
| Interoceptive awareness as measured by the Brief Multidimensional Assessment of Interoceptive Awareness, version 2 (Brief MAIA-2) [(20)](https://paperpile.com/c/0oMQDJ/KQVSX) | The Multidimensional Assessment of Interoceptive Awareness (MAIA-II) is a 37-item self-report questionnaire measuring 8 factors of interoceptive awareness: Noticing, Not-Distracting, Not-Worrying, Attention Regulation, Emotional Awareness, Self-Regulation, Body Listening and Trust. Statements are rated using a 5 point Likert scale, with scores ranging from 0 “Never” to 5 “Always”, with some items using reverse scoring. The outcome of each subscale is obtained by taking the average score of the items in each subscale, with higher scores indicating a better outcome. | t_1_,t_2_,t_3_,t_4,_t_5_,t_6_,t_7_ |
| Felt Sense of Anomaly as measured by the Short Form Černis Felt Sense of Anomaly Scale (ČEFSA) [(21)](https://paperpile.com/c/0oMQDJ/YqyyY) | The Short Form Černis Felt Sense of Anomaly Scale (ČEFSA) measures felt sense of anomaly, as a type of common dissociative experience. Possible scores range from 0 to 56, with higher scores indicating higher severity of dissociative experience. The severity categories are Average (0-28), Elevated (29-38), Moderately severe (39-49) and Severe (49-56). | t_1_,t_2_,t_3_,t_4,_t_5_,t_6_,t_7_ |
| Materialism as measured by the 9-item Material Values Scale (MVS) [(22)](https://paperpile.com/c/0oMQDJ/2rTec) | The Material Values Scale (MVS) measures the level of importance that one ascribes to material goods. Scores range from 9 to 45 with higher scores indicating material values having a higher importance. | t_1_,t_4,_t_5_,t_6_,t_7_ |
| Emotional breakthroughs as measured by the Emotional Breakthrough Inventory (EBI) [(23)](https://paperpile.com/c/0oMQDJ/AN31c) | The Emotional Breakthrough Inventory (EBI) measures the extent and quality of emotional breakthroughs experienced during a psychedelic session. Possible scores range from 0 to 100, with higher scores indicating a better outcome. | t_3_ |
| Geneva Emotional Music Scale - 9 (GEMS-9) | The GEMS-9 is a validated scale used to assess complex emotional responses to music. Participants rate the intensity of nine music-evoked emotions: wonder, transcendence, tenderness, peacefulness, nostalgia, joyful activation, power, sadness, and tension. Each item is rated on a 6-point Likert scale ranging from 0 (Not at all) to 5 (Very much). Higher scores indicate stronger emotional resonance with the musical experience. The GEMS-9 was administered retrospectively for each psilocybin EEG-ECG scan and also for the entire psilocybin journey. | t_3_ |
| Psychedelic Music Questionnaire - Short Form (SF) | The PMQ-SF is an adapted self-report scale designed to assess participants’ subjective experience of music during psychedelic sessions. It includes items evaluating emotional resonance, perceived support, personal meaning, openness to the music, and the degree to which the music shaped or guided the psychedelic experience. Items are rated on Likert scales ranging from 1 (Not at all) to 5 (Extremely), with higher scores reflecting greater perceived musical significance. The PMQ-SF was administered retrospectively for each psilocybin EEG-ECG scan and also for the entire psilocybin journey. | t_3_ |
| Affect as measured by the Positive and Negative Affect Schedule (PANAS) [(24)](https://paperpile.com/c/0oMQDJ/uYI9L) | The Positive and Negative Affect Schedule (PANAS) measures self-reported affect in two dimensions: positive and negative affect. In each dimension, possible scores range between 10 and 50, higher scores indicating higher positive/negative affect. | t_3_ |
| Sobriety as measured by the Drug Effects Questionnaire (DEQ) [(25)](https://paperpile.com/c/0oMQDJ/HG3HW) | The Drug Effects Questionnaire (DEQ) measures acute subjective responses to substances through 5 constructs (FEEL, HIGH, DISLIKE, LIKE, and MORE). Each construct is measured on a 100mm visual analogue scale. Participants are deemed as sober and ready to discharge if they score under 50 in the FEEL, HIGH and DISLIKE items. | t_3_ |
| Sobriety as measured by the 6-item Dissociative Symptom Scale (CADSS-6) [(26)](https://paperpile.com/c/0oMQDJ/cj0aK) | The 6-item Dissociative Symptom Scale (CADSS-6) assesses dissociation as an emergent adverse event during drug dosing. Potential scores range between 0 and 20, with higher scores indicating more severe dissociative symptoms. This questionnaire is administered before dosing (as baseline) and after dosing (to provide a sobriety measure). Participants are deemed as sober and ready to discharge if their post-dosing score is below 4 and the score increase from baseline is less than 3. | t_3_ |
| Saliva samples for cortisol analysis | Non-invasive sampling to assess changes in cortisol, a biomarker of HPA-axis activation. Allows exploration of stress physiology across the study, including sex-based differences (e.g., hormonal contraceptive use and menstrual phase). | t_1_,t_2,_t_3_,t_4_ |
| WHOOP wearable devices for continuous biometric tracking | Continuous collection of HR, HRV, temperature, and sleep timing to evaluate stress-related physiological changes over time and assess whether meditation, psilocybin, or sex-related factors contribute to HPA-axis variability. | t_1_-t_4_ |

**S6 DIPP intervention standardised instructions**

"Before we go into the details of how the DIPP platform works, I just want to say a bit about where it comes from. DIPP was developed by a team of expert researchers and clinicians, working in close collaboration with people who’ve actually taken part in psychedelic therapy. So, it’s been shaped not just by scientific evidence, but also by real experiences and feedback from participants like you. The whole programme is designed with one purpose: to support you in getting the most out of your psilocybin session. It’s based on research into what helps people feel prepared, grounded, and open to the process - so every part of it has been thoughtfully included to help you on that journey. In your Participant Folder there is a DIPP manual with step-by-step instructions for you to read through this evening, but for now I'll run you through the basics.

So, the DIPP platform is a 21-day programme designed to help you prepare for your psilocybin session. Each day, you’ll be guided through three steps:

- Daily morning practice
- A short mood check-in
- A journal prompt.

Altogether, this can take up to about an hour, so we recommend doing it first thing in the morning if you can.

When you open the app, you’ll see what day you’re on. Just tap that day to begin. Once you complete the morning practice, the app will automatically move you on to the mood check-in and then the journal. As you complete each part, it will be marked in green, so you can easily track your progress. It’s important to know that you can’t go back and catch up on missed days. So if you skip a day, unfortunately, that content will stay locked. That’s why it’s really important to check in every day - otherwise, you’ll miss out on what was included in the programme for that day.

In addition to the daily practices, each week you’ll unlock two extra activities. These are designed to further support your preparation. They become available at the start of each week, and you can complete them in your own time. Just make sure to click to mark them as completed once you’ve done them. One thing to be aware of: these weekly activities are found inside that day’s content, not on the main dashboard, so they can be easy to miss if you’re not looking out for them. You’ll find a booklet in your Participant Folder that gives you step-by-step instructions on how to use the DIPP platform. There’s also a reminder in there about where to find the weekly tasks. So when you get home, please take a few minutes to read through that guide carefully - it’ll make everything much easier."

As I mentioned earlier, you’ll find very detailed, step-by-step instructions in the DIPP Handbook, which is included in your Participant Folder. That guide walks you through exactly how to use the platform, so if you ever feel unsure, you can refer back to it at any time.

In your folder, you’ll also find your login details for the DIPP platform. Your username is the same as your usual participant ID, and we’ve provided a password for you to use as well. These are printed clearly in your folder so you can keep them handy.

Do you have any questions?"

**S7 Overview of DIPP-Bot in the study**

**Purpose**

The ‘DIPPbot’ serves as an interactive experience sampling tool for the trial, developed using Telegram Bot API and hosted via OneReachAI services. The bot facilitates two core study components: (1) DIPP Intervention: a 21-day preparatory program, and (2) Thought Sampling: real-time data collection on participants' mental states.

**System overview**

The rule-based chatbot operates through automated flows managing user interactions, notifications, and data collection. Participants register using study-assigned IDs, receive scheduled notifications, and submit voice notes and responses that are securely stored on AWS servers. The system includes (1) automated scheduling: daily DIPP reminders at 6:00 AM, thought samples prompts at random times in two intervals (first between 11:00 AM and 4:00 PM, and second between 4:00 PM at 9:00 PM), and (2) automatic transcription, using AssemblyAI API.

**Data collection schedule**

- **DIPP Intervention:** 21 days leading up to dosing day
- **Thought sampling**
  - Pre-dosing: 10 days before psilocybin administration
  - Post-dosing: 10 days after psilocybin administration
  - Follow-up periods: 7 days each at 1-, 3-, 6-, and 9-months post-dosing
- **Additional reminders**
  - Reminder for participants to collect a cortisol sample on specified days

**DIPP-Bot architecture**

The diagram below illustrates the architecture of the DIPPbot, outlining the different flows that manage user interactions and notifications. Each node in the diagram represents a distinct flow or process, and the arrows indicate the direction and sequence of actions. Participant IDs are uploaded to the bot database together with respective study visit dates during onboarding. The architecture is divided into two main sections: *User Initiated* and *Scheduled* flows. User-initiated flows are triggered when a participant interacts with the chatbot, such as starting the bot, sending a voice note, or responding to a message. The Telegram Intake Flow manages these initial interactions and directs users based on their status (new or returning). New users go through a registration process, while returning users may proceed directly to the Thought Sampling flow. Scheduled flows, on the other hand, operate automatically at predefined times. For instance, the Schedule Daily Notifications and Schedule Thought Sampling Notifications flows trigger daily and twice-daily prompts, respectively, guiding participants to complete preparatory tasks or submit thought samples. These flows ensure data consistency, manage user engagement, and handle data storage in a structured manner throughout the study.


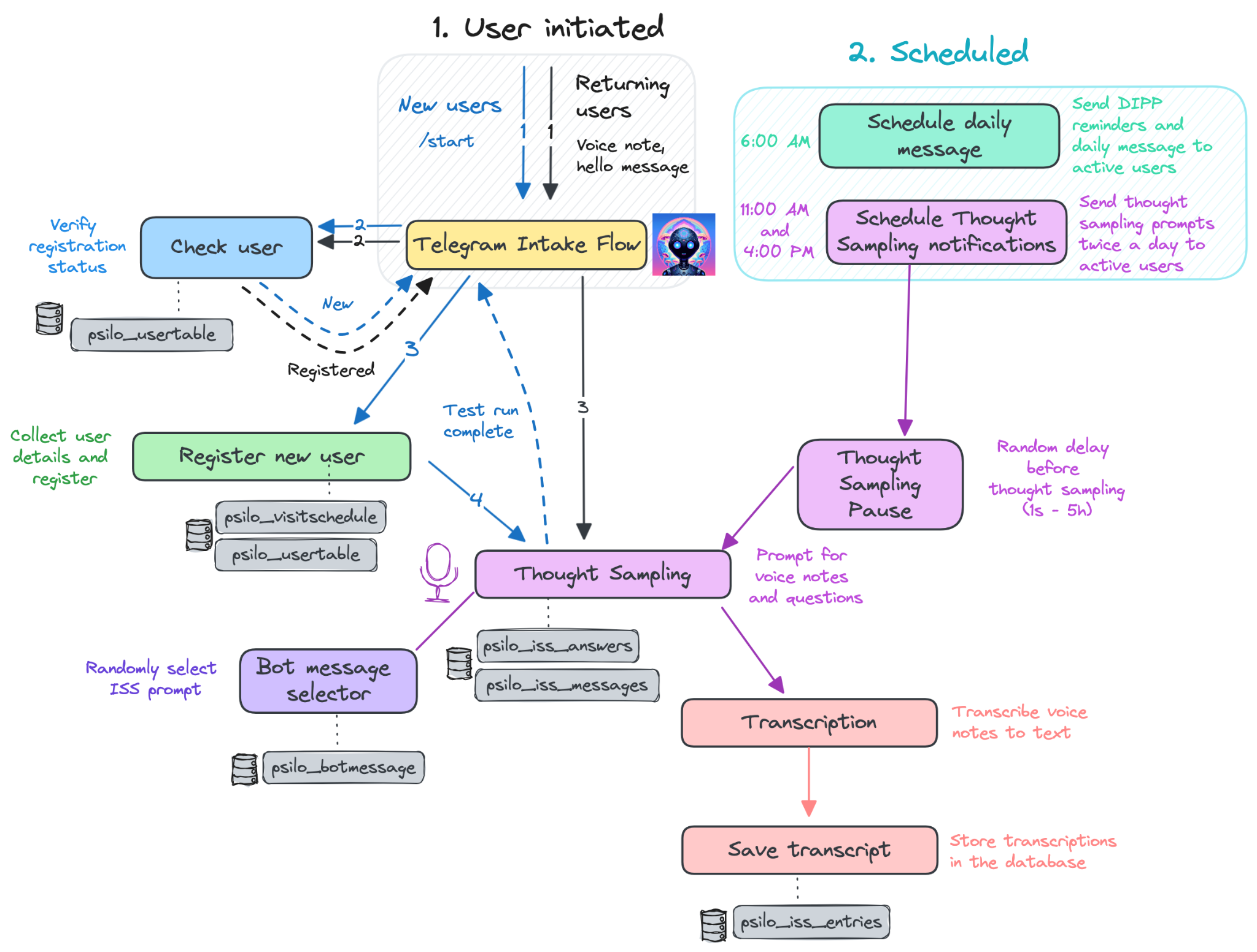


**Figure S7-1.** Overview flowchart of DIPPbot Architecture, illustrating the user-initiated and scheduled flows that manage participant interactions, notifications, and data processing.

**Key Prompts and Messages**

| **Category** | **Prompt** |
| --- | --- |
| **Daily reminder** | Welcome on Day [X]!   [A] It's time for your daily DIPP morning practice.  Please follow the link below and make sure to complete your daily exercises before 11:00.  <https://www.dipp.uk/>  [B] You’ll receive 2 thought sampling notifications later today. When you do, try to send a voice note within an hour!  [C] Remember to take your cortisol sample today.  ​(No need to reply to this message). |
| **Thought sampling prompt** | It's time for a bit of thought sampling. Ready to dive into your mind? Let's capture the thoughts that are passing through right now—in this very moment. ​​  ​ Here are some prompts to guide you:​  ​About: What are these thoughts centered around? Are they focused on a particular subject or theme?​  ​People, Places, Things, Ideas: Who or what is featuring in your thoughts? Any specific ideas or visions?​  ​Emotions: What emotions are intertwined with these thoughts? Are they bringing joy, anxiety, curiosity, or a mix of feelings?​  ​Origins: What sparked these thoughts? An event, a conversation, or something else?​  ​​  ​Take your time to ponder these aspects. When you're ready, record a voice note. Try to speak for ~1 minute, going through the points above. |

**Table S7-1. Key prompts and messages.** For the Daily reminder, presentation of [A], [B], or [C] is conditional on study date relative to participant’s dosing (as outlined in Data collection schedule). The thought sampling prompt is delivered with each notification of this kind and includes a brief guidance that precedes the participant’s 1-minute voice note. Participants are requested to submit the voice note within one hour of receiving this notification.

| **Adapted from** | **Purpose** | **Question code** | **Item** |
| --- | --- | --- | --- |
| Descriptive experience sampling (DES) method  [(27)](https://paperpile.com/c/0oMQDJ/tFxix) | Thought type classification | A01 | Which thought type were you having? Choose the best matching. ​  ​​1= Inner speaking (the experience of mentally speaking to yourself in your own voice, without making sound or moving your mouth)​  ​2= Inner seeing (the experience of mentally seeing images, without using your eyes)​  ​3= Unsymbolised thinking (the experience of thinking that is not accompanied by any mental words, mental images or any other symbols, it is just direct abstract thought)​  ​4= Sensory awareness (focused on the experience of a sensation itself, not for any purpose)​  ​5= Feelings (the experience of emotion) |
| Multidimensional experience-sampling (MDES) questions [(28)](https://paperpile.com/c/0oMQDJ/xu5z4) | Thought characteristics | B01 | My thoughts were focused on an external task or activity (1-Not at all, 10-Completely) |
|  |  | B02 | My thoughts involved future events (1-Not at all, 10-Completely) |
|  |  | B03 | My thoughts involved past events (1-Not at all, 10-Completely) |
|  |  | B04 | My thoughts involved myself (1-Not at all, 10-Completely) |
|  |  | B05 | My thoughts involved other people (1-Not at all, 10-Completely) |
|  |  | B06 | My thoughts involved images (1-Not at all, 10-Completely) |
|  |  | B07 | My thoughts were detailed and specific (1-Not at all, 10-Completely) |
|  |  | B08 | I was thinking about solutions to problems (or goals) (1-Not at all, 10-Completely) |
|  |  | B09 | My thoughts were intrusive (1-Not at all, 10-Completely) |
|  |  | B10 | My thoughts contained information I already knew (e.g., knowledge or memories) (1-Not at all, 10-Completely) |
|  |  | B11 | I was absorbed in the contents of my thoughts (1-Not at all, 10-Completely) |
|  |  | B12 | My thoughts were distracting me from what I am doing (1-Not at all, 10-Completely) |
|  |  | B13 | My thoughts involved words (1-Not at all, 10-Completely) |
|  |  | B14 | My thoughts involved sounds (1-Not at all, 10-Completely) |
|  |  | B15 | The emotion of my thoughts was (1- Negative, 10-Positive) |
|  |  | B16 | My thoughts were (1-Spontaneous, 10-Deliberate) |
|  | Social context | B17 | Were you alone or with other people (physically and not virtually) just before taking this survey?​  ​​1= Alone,​  ​2= Around people but not interacting, ​  ​3= Around people and interacting |
|  |  | B18 | Virtually, were you alone or with other people just before taking this survey?​  ​​1= Alone​  ​2= Around people but not interacting with them (e.g., reading messages but not replying, being on a video call but not talking/participating etc)​  ​3= Around people virtually and interacting with them (e.g., text, instant messaging, calling, or video calling etc.) |
|  | Location and activity | B19 | WHERE were you just before taking this survey? ​  ​Type your answer (e.g., home, work, park, gym, restaurant). |
|  |  | B20 | What were you DOING just before taking this survey? ​  ​Type your answer (e.g. exercising, reading, cooking, working, etc) |

**Table S7-2. Experience sampling questions.** The questions are presented consequentially after the thought sample is submitted. The questions include *thought type classification* (5 categories: inner speaking, inner seeing, unsymbolized thinking, sensory awareness, feelings), 16 Likert scale questions (1-10) covering *thought characteristics* (focus, detail, temporal orientation, emotional valence, etc.), *social context questions* (physical/virtual presence of others), and *location and activity* open-text question.

**References**

1. [Sekhon M, Cartwright M, Francis JJ. Development of a theory-informed questionnaire to assess the acceptability of healthcare interventions. BMC Health Serv Res. 2022 Mar 1;22(1):279.](http://paperpile.com/b/0oMQDJ/7oqrq)

2. [Brooke J. SUS: A “quick and dirty” usability scale. Usability evaluation in industry. 1996 Jun 11;207–12.](http://paperpile.com/b/0oMQDJ/mHb9y)

3. [Stoyanov SR, Hides L, Kavanagh DJ, Zelenko O, Tjondronegoro D, Mani M. Mobile app rating scale: a new tool for assessing the quality of health mobile apps. JMIR Mhealth Uhealth. 2015 Mar 11;3(1):e27.](http://paperpile.com/b/0oMQDJ/38YS7)

4. [McAlpine RG, Blackburne G, Kamboj SK. Development and psychometric validation of a novel scale for measuring “psychedelic preparedness.” Sci Rep. 2024 Feb 8;14(1):1–15.](http://paperpile.com/b/0oMQDJ/0CZdo)

5. [Studerus E, Gamma A, Vollenweider FX. Psychometric evaluation of the altered states of consciousness rating scale (OAV). PLoS One. 2010 Aug 31;5(8):e12412.](http://paperpile.com/b/0oMQDJ/LP6wn)

6. [Barrett FS, Bradstreet MP, Leoutsakos JMS, Johnson MW, Griffiths RR. The Challenging Experience Questionnaire: Characterization of challenging experiences with psilocybin mushrooms. J Psychopharmacol. 2016 Dec;30(12):1279–95.](http://paperpile.com/b/0oMQDJ/nXd62)

7. [Tennant R, Hiller L, Fishwick R, Platt S, Joseph S, Weich S, et al. The Warwick-Edinburgh Mental Well-being Scale (WEMWBS): development and UK validation. Health Qual Life Outcomes. 2007 Nov 27;5:63.](http://paperpile.com/b/0oMQDJ/WZly9)

8. [Devilly GJ, Borkovec TD. Psychometric properties of the credibility/expectancy questionnaire. J Behav Ther Exp Psychiatry. 2000 Jun;31(2):73–86.](http://paperpile.com/b/0oMQDJ/S6zd)

9. [Nummenmaa L, Glerean E, Hari R, Hietanen JK. Bodily maps of emotions. Proc Natl Acad Sci U S A. 2014 Jan 14;111(2):646–51.](http://paperpile.com/b/0oMQDJ/1AFRQ)

10. [Dijkstra N, Mazor M, Fleming SM. Confidence ratings do not distinguish imagination from reality. J Vis. 2024 May 1;24(5):13.](http://paperpile.com/b/0oMQDJ/TJxMR)

11. [Feldman G, Hayes A, Kumar S, Greeson J, Laurenceau JP. Mindfulness and emotion regulation: The development and initial validation of the cognitive and affective mindfulness scale-revised (CAMS-R). J Psychopathol Behav Assess. 2007 Jul 13;29(3):177–90.](http://paperpile.com/b/0oMQDJ/dDojl)

12. [Gu J, Baer R, Cavanagh K, Kuyken W, Strauss C. Development and Psychometric Properties of the Sussex-Oxford Compassion Scales (SOCS). Assessment. 2020 Jan;27(1):3–20.](http://paperpile.com/b/0oMQDJ/kg37z)

13. [Benoy C, Knitter B, Knellwolf L, Doering S, Klotsche J, Gloster AT. Assessing psychological flexibility: Validation of the Open and Engaged State Questionnaire. Journal of Contextual Behavioral Science. 2019 Apr 1;12:253–60.](http://paperpile.com/b/0oMQDJ/erhKC)

14. [Kroenke K, Spitzer RL, Williams JBW. The PHQ-9. J Gen Intern Med. 2001 Sep;16(9):606–13.](http://paperpile.com/b/0oMQDJ/cNV9M)

15. [Löwe B, Decker O, Müller S, Brähler E, Schellberg D, Herzog W, et al. Validation and standardization of the Generalized Anxiety Disorder Screener (GAD-7) in the general population. Med Care. 2008 Mar;46(3):266–74.](http://paperpile.com/b/0oMQDJ/GQccF)

16. [Treynor W. Rumination reconsidered: A psychometric analysis. Cognit Ther Res. 2003;27(3):247–59.](http://paperpile.com/b/0oMQDJ/MjFG)

17. [Alderson-Day B, Mitrenga K, Wilkinson S, McCarthy-Jones S, Fernyhough C. The varieties of inner speech questionnaire - Revised (VISQ-R): Replicating and refining links between inner speech and psychopathology. Conscious Cogn. 2018 Oct;65:48–58.](http://paperpile.com/b/0oMQDJ/KIW8)

18. [Heavey CL, Moynihan SA, Brouwers VP, Lapping-Carr L, Krumm AE, Kelsey JM, et al. Measuring the Frequency of Inner-Experience Characteristics by Self-Report: The Nevada Inner Experience Questionnaire. Front Psychol. 2018;9:2615.](http://paperpile.com/b/0oMQDJ/gfVh)

19. [Ehring T, Zetsche U, Weidacker K, Wahl K, Schönfeld S, Ehlers A. The Perseverative Thinking Questionnaire (PTQ): validation of a content-independent measure of repetitive negative thinking. J Behav Ther Exp Psychiatry. 2011 Jun;42(2):225–32.](http://paperpile.com/b/0oMQDJ/tXbLx)

20. [Rogowska AM, Tataruch R, Klimowska K. Validation of the shortened 24-item multidimensional assessment of interoceptive awareness, version 2 (Brief MAIA-2). Sci Rep. 2023 Dec 2;13(1):21270.](http://paperpile.com/b/0oMQDJ/KQVSX)

21. [Černis E, Loe BS, Lofthouse K, Waite P, Molodynski A, Ehlers A, et al. Measuring dissociation across adolescence and adulthood: developing the short-form Černis Felt Sense of Anomaly scale (ČEFSA-14). Behav Cogn Psychother. 2024 Mar;52(2):163–77.](http://paperpile.com/b/0oMQDJ/YqyyY)

22. [Richins ML. The material values scale: Measurement properties and development of a short form. J Consum Res. 2004 Jun;31(1):209–19.](http://paperpile.com/b/0oMQDJ/2rTec)

23. [Roseman L, Haijen E, Idialu-Ikato K, Kaelen M, Watts R, Carhart-Harris R. Emotional breakthrough and psychedelics: Validation of the Emotional Breakthrough Inventory. J Psychopharmacol. 2019 Sep;33(9):1076–87.](http://paperpile.com/b/0oMQDJ/AN31c)

24. [Watson D, Clark L, Tellegen A. Development and validation of brief measures of positive and negative affect: the PANAS scales. J Pers Soc Psychol. 1988 May 30;54(6):1063–70.](http://paperpile.com/b/0oMQDJ/uYI9L)

25. [Morean ME, de Wit H, King AC, Sofuoglu M, Rueger SY, O’Malley SS. The drug effects questionnaire: psychometric support across three drug types. Psychopharmacology (Berl). 2013 May;227(1):177–92.](http://paperpile.com/b/0oMQDJ/HG3HW)

26. [Rodrigues NB, McIntyre RS, Lipsitz O, Lee Y, Cha DS, Shekotikhina M, et al. A simplified 6-Item clinician administered dissociative symptom scale (CADSS-6) for monitoring dissociative effects of sub-anesthetic ketamine infusions. J Affect Disord. 2021 Mar 1;282:160–4.](http://paperpile.com/b/0oMQDJ/cj0aK)

27. [Hurlburt RT, Heavey CL. Exploring Inner Experience. John Benjamins; 2006.](http://paperpile.com/b/0oMQDJ/tFxix)

28. [Mckeown B, Poerio GL, Strawson WH, Martinon LM, Riby LM, Jefferies E, et al. The impact of social isolation and changes in work patterns on ongoing thought during the first COVID-19 lockdown in the United Kingdom. Proc Natl Acad Sci U S A [Internet]. 2021 Oct 5;118(40). Available from:](http://paperpile.com/b/0oMQDJ/xu5z4) <http://dx.doi.org/10.1073/pnas.2102565118>
