# Supplementary material for: Digital Intervention for Psychedelic Preparation (DIPP): protocol for a randomised controlled feasibility trial comparing meditation- and music-based programmes in healthy volunteers
Source: BMJ Open. 2026 Mar 12;16(3):e107512. doi: 10.1136/bmjopen-2025-107512 (PMC12983862; doi:10.1136/bmjopen-2025-107512)
Supplement: Supplementary file 2 [file bmjopen-2025-107512.reviewer_comments.pdf]

PEER REVIEW HISTORY

BMJ Open publishes all reviews undertaken for accepted manuscripts. Reviewers are asked to complete a checklist review form and are provided with free text boxes to elaborate on their assessment. These free text comments are reproduced below.

ARTICLE DETAILS

Title (Provisional)

Digital Intervention for Psychedelic Preparation (DIPP): Protocol for a randomised controlled feasibility trial comparing meditation and non-meditation programs in healthy volunteers

Authors

McAlpine, Rosalind; Jaglinska, Magdalena; Jedlovszky, Krisztina; Kuc, Joanna; Castro, Ariel; Piot, Alexandre; Timmermann, Christopher; Skipper, Jeremy I; Sacchet, Matthew D; Kamboj, Sunjeev K

VERSION 1 - REVIEW

**Reviewer**	**1**
**Name**	**Capone, Christy**
**Affiliation**	**Providence Veterans Affairs Medical Center, Providence, RI 02908, USA.**
**Date**	**11-Nov-2025**
**COI**	**None**

Thank you for the opportunity to review this manuscript. This paper details the protocol of an upcoming randomized, controlled feasibility study comparing two digital-based platforms aiming to prepare participants for psychedelic experiences. The sample will be comprised of 40 healthy individuals; thus, this is a nonclinical sample. All participants will engage with a 21-day web-based app and be randomized to either a meditation-based program or no meditation. Following the digital preparation, all participants will engage in a supervised psilocybin session and will complete follow-up assessments 2 weeks post-session, and 3, 6, and 9 months later. Primary outcomes for the study are focused on feasibility, while secondary outcomes include further measures of feasibility and acceptability, as well as efficacy of this approach to preparation for psychedelic experiences. Exploratory outcomes include assessment of cognitive, psychological, and physiological measures.

The study is novel and timely given the surge in research and popular interest in psychedelic treatment approaches. The reasoning behind a web-based approach to preparation makes sense from a scalability standpoint, though it is unclear how feasible this approach would be with a clinical sample. The stakeholder involvement is a strength of this research. However, more detail is

needed regarding various aspects of the protocol, including the imaging component, the DIPP-bot, and some of the study procedures (e.g., the informed consent process). Also, the various design elements (imaging, chatbot, biometric data, cognitive tasks) need to be outlined from the start along with supporting rationale and the empirical questions that this study aims to answer. These issues are all addressable and a revised version could make a nice addition to the literature.

The following are comments/questions that I hope will serve to clarify and strengthen the manuscript:

1. It would help to clarify how the two conditions could be matched on dose (time) since one group does not receive the bulk of the content (meditation). It seems to me that there must be a discrepancy between the two conditions.

2. The imaging component of the trial should be clarified at the outset. It first comes up as part of the eligibility criteria, but it is not clear where this is coming from. It is mentioned again on page 12, but no rationale is provided for the fMRI procedures.

3. Please clarify if some mental health conditions are allowed since the protocol specifically mentions some (e.g. bipolar disorder) but not others (e.g. PTSD, substance use).

4. The rationale and protocol for the chatbot is not very well developed. It comes across as an add on component, but it is not clear what it adds to the protocol. What is the purpose of the daily "voice note samples?"

5. Please describe the informed consent procedures. It appears that the study involves a form of deception in that participants are not informed that there are different versions of the intervention. Are they made aware that they will be randomized to one of two conditions? Who is conducting the informed consent process?

6. Similarly, the use of wearable devices is mentioned on page 11, but the rationale and specifics of what data is being collected is not described. It would help of all of the elements of the study design were mentioned at the beginning, ideally in terms of how they map on to unaddressed questions in the literature and this study's aims and objectives.

7. On page 14, the authors state that a DMC is not required as this study "does not involve an investigational medicinal product." However, in the section above ("IMP management)" it clearly states that psilocybin is an IMP. Please clarify this discrepancy.

8. It is surprising that a psilocybin study would be designated as "low risk." Is this related to the sample being comprised of what would be considered healthy volunteers?

9. It seems that citations are missing for the measures used in the study.

10. How long is the integration session conducted two weeks post-dosing? Who does the participant meet with for this session?

11. As the study emphasizes the web-based preparation platform, it was not clear how much interaction the participant has with the "guides." On page 15, it seems that the guides meet with the participant at baseline to help prepare them for the experience. How long is this meeting? It

would help to clarify sooner in the manuscript how often and for how long the participants have contact with their "guides." Perhaps a flow chart or other figure would help to illustrate some of the points raised here regarding the need for more detail and clarification.

12. It appears that the consent form may have already been approved for this study, but the language used in it is fairly advanced, and not at the 8th grade reading level that is typically recommended.

VERSION 1 - AUTHOR RESPONSE

Reviewer: 1

Dr. Christy Capone, Providence Veterans Affairs Medical Center, Providence, RI 02908, USA., Brown University

Comments to the Author:

Thank you for the opportunity to review this manuscript. This paper details the protocol of an upcoming randomized, controlled feasibility study comparing two digital-based platforms aiming to prepare participants for psychedelic experiences. The sample will be comprised of 40 healthy individuals; thus, this is a nonclinical sample. All participants will engage with a 21-day web-based app and be randomized to either a meditation-based program or no meditation. Following the digital preparation, all participants will engage in a supervised psilocybin session and will complete follow-up assessments 2 weeks post-session, and 3, 6, and 9 months later. Primary outcomes for the study are focused on feasibility, while secondary outcomes include further measures of feasibility and acceptability, as well as efficacy of this approach to preparation for psychedelic experiences. Exploratory outcomes include assessment of cognitive, psychological, and physiological measures. The study is novel and timely given the surge in research and popular interest in psychedelic treatment approaches. The reasoning behind a web-based approach to preparation makes sense from a scalability standpoint, though it is unclear how feasible this approach would be with a clinical sample. The stakeholder involvement is a strength of this research. However, more detail is needed regarding various aspects of the protocol, including the imaging component, the DIPP-bot, and some of the study procedures (e.g., the informed consent process). Also, the various design elements (imaging, chatbot, biometric data, cognitive tasks) need to be outlined from the start along with supporting rationale and the empirical questions that this study aims to answer. These issues are all addressable and a revised version could make a nice addition to the literature.

- Response: Thank you for your thoughtful and constructive review. We appreciate your recognition of the study's novelty and timeliness, and your acknowledgment of the stakeholder involvement as a strength. We have carefully addressed each of your specific comments. In particular, we have added clearer rationale and earlier introduction of the imaging and physiological monitoring components, clarified the informed consent process (including our approach to incomplete disclosure), provided more detail on guide contact and study procedures, and ensured consistency between the protocol and trial registry. We agree that applicability to clinical samples remains an open question and have noted this as a limitation. We hope the revised manuscript now provides the clarity and detail needed, and we thank you for helping strengthen this work.

1. **It would help to clarify how the two conditions could be matched on dose (time) since one group does not receive the bulk of the content (meditation). It seems to me that there must be a discrepancy between the two conditions.**
    - Response: Thank you for this helpful comment. We have now clarified the duration matching between conditions by adding the following text to the 'Trial objectives' section: *'Importantly, the two conditions are fully matched on exposure time: the DIPP-NM audio presents the same background music for the full session duration, with only the guided meditation instructions removed'.*
2. **The imaging component of the trial should be clarified at the outset. It first comes up as part of the eligibility criteria, but it is not clear where this is coming from. It is mentioned again on page 12, but no rationale is provided for the fMRI procedures.**
    - Response: Thank you for this suggestion. We have revised the Introduction to include a consolidated paragraph introducing all exploratory components, including the naturalistic fMRI. The text now clarifies the rationale (naturalistic paradigms offer enhanced ecological validity and can reveal changes in functional network organisation) and the empirical question (whether the intervention and psilocybin session are associated with whole-brain functional network changes). We also clarify that standard MRI eligibility criteria are included because participation requires two fMRI sessions.
3. **Please clarify if some mental health conditions are allowed since the protocol specifically mentions some (e.g. bipolar disorder) but not others (e.g. PTSD, substance use).**
    - Response: Thank you for this important point regarding clarity of psychiatric exclusion criteria. We have revised the eligibility criteria section to improve transparency. The text now specifies that individuals will be excluded if they have any current or past diagnosis of a psychiatric disorder (including PTSD, substance use disorders, etc.), with a narrow exception for individuals with a history of a mood disorder provided they have been in stable remission for at least five years and are assessed as low risk by the clinical team. Psychotic and bipolar disorders remain absolute exclusions.
4. **The rationale and protocol for the chatbot is not very well developed. It comes across as an add on component, but it is not clear what it adds to the protocol. What is the purpose of the daily "voice note samples?"**
    - Response: Thank you for this feedback. We have expanded the rationale for the DIPP-Bot in the Introduction. The revised text clarifies that thought sampling complements traditional questionnaires by capturing momentary inner experience rather than retrospective aggregated reports. Voice notes specifically capture both semantic content and paralinguistic features (e.g., speech rate, pitch variability) that may index emotional and cognitive states - allowing examination of how inner experience shifts across the psilocybin experience. Full methodological details are provided in Supplementary S7.
5. **Please describe the informed consent procedures. It appears that the study involves a form of deception in that participants are not informed that there are different versions of the intervention. Are they made aware that they will be randomized to one of two conditions? Who is conducting the informed consent process?**
    - Response: Thank you for raising this important point. We have clarified the informed consent procedures. Participants are not informed that the study involves

randomisation or that there are different versions of the intervention.

sDuring consent, they are told they will complete a 21-day digital preparation programme involving daily audio-based activities; no reference is made to different conditions. This incomplete disclosure is necessary to control for expectancy effects that could otherwise confound results. Participants are debriefed at study completion (t7) and informed of the study design and their allocated condition. Informed consent is obtained remotely by trained research staff. This procedure was reviewed and approved by the UCL Research Ethics Committee.

6. **Similarly, the use of wearable devices is mentioned on page 11, but the rationale and specifics of what data is being collected is not described. It would help of all of the elements of the study design were mentioned at the beginning, ideally in terms of how they map on to unaddressed questions in the literature and this study's aims and objectives.**
    - Response: Thank you for this helpful suggestion. We have added a consolidated paragraph in the Introduction outlining all exploratory study components (thought sampling, fMRI, physiological monitoring, cognitive tasks), the data being collected, and the empirical questions each addresses. A complete overview of all outcome measures is provided in Supplementary S4 (SPIRIT table) and S5. We believe this revision ensures all design elements are introduced at the outset with supporting rationale.
7. **On page 14, the authors state that a DMC is not required as this study "does not involve an investigational medicinal product." However, in the section above ("IMP management)" it clearly states that psilocybin is an IMP. Please clarify this discrepancy.**
    - Response: Thank you for identifying this discrepancy. The statement that the study *'does not involve an investigational medicinal product*' was an error. We have revised the Data Monitoring section to remove this incorrect claim. The rationale for not convening an independent DMC is now accurately stated: the decision is based on the small sample size (n=40), single-dose administration, recruitment of healthy volunteers rather than a clinical population, the absence of planned interim efficacy analyses, and the established safety profile of psilocybin at this dose in supervised research settings with medical oversight. This approach is consistent with proportionate risk-based monitoring guidance for early-phase experimental medicine studies.
8. **It is surprising that a psilocybin study would be designated as "low risk." Is this related to the sample being comprised of what would be considered healthy volunteers?**
    - Response: Thank you for raising this point. We agree that 'low risk' is not an appropriate characterisation for a psilocybin study and have removed this language from the manuscript. While the study recruits healthy volunteers and employs comprehensive screening, medical supervision, and standardised safety protocols, we recognise that psilocybin administration carries inherent risks. The revised text now describes monitoring procedures as *'proportionate to study risk'* rather than characterising the study itself as low risk.

9. **It seems that citations are missing for the measures used in the study.**
    - Response: Thank you for noting this omission. We have added citations for all measures used in the study.
10. **How long is the integration session conducted two weeks post-dosing? Who does the participant meet with for this session?**
    - Response: Thank you for this query. The integration session at two weeks post-dose (t4) lasts 30-60 minutes and is conducted by the same guides who accompanied the participant during the dosing session. We have clarified this in the revised text.
11. **As the study emphasizes the web-based preparation platform, it was not clear how much interaction the participant has with the "guides." On page 15, it seems that the guides meet with the participant at baseline to help prepare them for the experience. How long is this meeting? It would help to clarify sooner in the manuscript how often and for how long the participants have contact with their "guides." Perhaps a flow chart or other figure would help to illustrate some of the points raised here regarding the need for more detail and clarification.**
    - Response: Thank you for this helpful suggestion. We have revised the Preparation section to clarify the frequency and duration of guide contact. The revised text now explicitly states that direct contact with guides is intentionally limited, with the majority of preparation delivered remotely via the DIPP platform. In-person guide contact comprises three touchpoints: baseline preparation (~30 minutes), pre-dosing preparation (~30-45 minutes), and supervision during dosing. Post-dosing contact includes an email check-in the day after dosing (with a call available on request) and an in-person integration session at two weeks (~30-60 minutes). We believe these clarifications adequately illustrate the study design.
12. **It appears that the consent form may have already been approved for this study, but the language used in it is fairly advanced, and not at the 8th grade reading level that is typically recommended.**
    - Response: Thank you for raising this point. The consent form has been reviewed and approved by the UCL Research Ethics Committee. Given the complexity of information that must be conveyed regarding psilocybin administration, potential risks, and study procedures, some advanced terminology is unavoidable. Importantly, informed consent is obtained verbally by trained research staff, with opportunities for participants to ask questions and verbal checks of understanding to ensure comprehension.

VERSION 2 - REVIEW

Reviewer **1**

Name **Capone, Christy**

Affiliation **Providence Veterans Affairs Medical Center, Providence, RI 02908, USA.**

Date **08-Feb-2026**

COI

Thank you for your thoughtful response to my previous comments. The revised manuscript attends to my questions/concerns and reads more clearly. I have no further comments and wish you well with the trial!
